# Strategies to adapt and implement health system guidelines and recommendations: a scoping review

**DOI:** 10.1186/s12961-022-00865-8

**Published:** 2022-06-15

**Authors:** Sydney Breneol, Janet A. Curran, Robert Marten, Kirti Minocha, Catie Johnson, Helen Wong, Etienne V. Langlois, Lori Wozney, C. Marcela Vélez, Christine Cassidy, Sanjay Juvekar, Melissa Rothfus, Lydia Aziato, Lisa Keeping-Burke, Samuel Adjorlolo, Daniel F. Patiño-Lugo

**Affiliations:** 1 School of Nursing, Faculty of Health, Dalhousie University, Halifax, Canada; 2 Strengthening Transitions in Care Lab, IWK Health Centre, 8th Floor Children’s Site, 5850/5980 University Ave, Halifax, NS B3K 6R8 Canada; 3 Alliance for Health Policy and Systems Research, World Health Organization, Geneva, Switzerland; 4 Faculty of Health, Dalhousie University, Halifax, Canada; 5 Partnership for Maternal, Newborn & Child Health (PMNCH), World Health Organization, Geneva, Switzerland; 6 Nova Scotia Health Authority Policy and Planning, Dartmouth, Canada; 7 Facultad de Medicina, Universidad de Antioquia, Medellín, Antioquia Colombia; 8 Vadu Rural Health Program, KEM Hospital Research Centre, Pune, India; 9 W.K. Kellogg Health Science Library, Dalhousie University, Halifax, Canada; 10 School of Nursing and Midwifery, University of Ghana, Legon, Accra, Ghana; 11 Department of Nursing & Health Sciences, University of New Brunswick, St. John, Canada; 12 Department of Mental Health Nursing, University of Ghana, Legon, Accra, Ghana

**Keywords:** Health systems, Global health, Scoping review, Implementation science, Evidence-informed guidelines

## Abstract

**Background:**

Evidence-based health system guidelines are pivotal tools to help outline the important financial, policy and service components recommended to achieve a sustainable and resilient health system. However, not all guidelines are readily translatable into practice and/or policy without effective and tailored implementation and adaptation techniques. This scoping review mapped the evidence related to the adaptation and implementation of health system guidelines in low- and middle-income countries.

**Methods:**

We conducted a scoping review following the Joanna Briggs Institute methodology for scoping reviews. A search strategy was implemented in MEDLINE (Ovid), Embase, CINAHL, LILACS (VHL Regional Portal), and Web of Science databases in late August 2020. We also searched sources of grey literature and reference lists of potentially relevant reviews. All findings were reported following the Preferred Reporting Items for Systematic Reviews and Meta-Analyses Extension for Scoping Reviews.

**Results:**

A total of 41 studies were included in the final set of papers. Common strategies were identified for adapting and implementing health system guidelines, related barriers and enablers, and indicators of success. The most common types of implementation strategies included education, clinical supervision, training and the formation of advisory groups. A paucity of reported information was also identified related to adaptation initiatives. Barriers to and enablers of implementation and adaptation were reported across studies, including the need for financial sustainability. Common approaches to evaluation were identified and included outcomes of interest at both the patient and health system level.

**Conclusions:**

The findings from this review suggest several themes in the literature and identify a need for future research to strengthen the evidence base for improving the implementation and adaptation of health system guidelines in low- and middle-income countries. The findings can serve as a future resource for researchers seeking to evaluate implementation and adaptation of health system guidelines. Our findings also suggest that more effort may be required across research, policy and practice sectors to support the adaptation and implementation of health system guidelines to local contexts and health system arrangements in low- and middle-income countries.

**Supplementary Information:**

The online version contains supplementary material available at 10.1186/s12961-022-00865-8.

## Background

Evidence-informed guidelines are pivotal to reforming healthcare and strengthening health systems for healthier communities worldwide [1, 2]. WHO conceptualizes guidelines as a set of evidence-informed recommendations related to practice, public health or policy for informing and assisting decision-makers (e.g. policy-makers, healthcare providers or patients) [3]. In contrast to clinical practice guidelines focused on the appropriateness of clinical care activities, health system guidelines outline the required system, policy and/or finance components recommended to address health challenges [4, 5].

Despite the rigorous systematic synthesis of current research evidence focused on the development of high-quality guidelines, not all guidelines are readily and directly translatable into practice and/or policy [6, 7]. According to Balas and Boren, the small proportion of published evidence (approximately 14%) that does translate into practice can take upwards of 17 years from start to finish [8, 9]. Understanding implementation and adaptation strategies that facilitate the uptake of evidence-informed guidelines and recommendations is an urgent research and policy priority [10–13]. Implementation strategies are often defined as “methods or techniques used to enhance the adaptation, implementation, and sustainability of a program or practice” [14]. Guideline adaptation strategies involve systematically modifying guidelines developed in a specific environment to be suitable for application in other contextual settings (e.g. organizational or cultural) [15].

A review of WHO guidelines by Wang et al. [16] revealed a lack of implementation strategies that were evidence-based and involved active techniques (e.g. workshops, evaluation surveys, training) within their relevant implementation sections. WHO is currently focused on enhancing the adaptability of guidelines [17] and integrating adaptation strategies into their implementation plans [18]. For successful uptake, even high-quality international guidelines require adapting and tailoring to local contexts or circumstances [19]. To help achieve success, the Alliance for Health Policy and Systems Research (a WHO-hosted partnership) created the Research to Enhance the Adaptation and Implementation of Health Systems Guidelines (RAISE) portfolio, which aims to support decision-making on policy and systems in six low- and middle-income countries (LMICs) [20]. However, much remains to be known about the factors and processes to enhance their adaptation and implementation [16, 20]. Additional evidence is needed to inform good practices, effective methods and evidence-based implementation and adaptation recommendations for the utilization of health system guidelines.

Neglecting to consider the interaction between contextual factors and guideline uptake is likely to lead to underperformance or failure [21–25]. It is important to recognize political, cultural and socioeconomic contexts and how these intersectional factors can influence health system guideline implementation and adaptation processes. Several methods have been derived for the selection and tailoring of implementation strategies to address these contextual needs [26]. Various taxonomies have been established as a means to better describe and categorize implementation strategies [27–33] and to conceptualize context to allow for the analysis of determinants (e.g. barriers and enablers) of implementation outcomes [34]. Frameworks have also been identified for adapting health-related guidelines, but often lack guidance on implementation [18, 35]. Therefore, the best methods for developing tailored implementation strategies and selecting adaptation frameworks remain to be identified [12, 18].

We conducted a preliminary search of PROSPERO, MEDLINE, the Cochrane Database of Systematic Reviews, and the Joanna Briggs Institute (JBI) Database of Systematic Reviews and Implementation Reports. No reviews were identified that addressed adapting and implementing health system guidelines in LMICs. The search revealed a related overview of systematic reviews examining the effects of implementation techniques for health system initiatives that were deemed relevant to low-income countries (LICs) [36]. Despite this review and the acknowledged contextual differences between LICs and high-income countries (HICs), the findings were derived primarily from studies conducted in HICs, leaving a significant gap in the literature examining any contextual nuances of implementation and adaptation of health system guidelines specifically in LMICs.

The objective of this scoping review is unique, as it provides an overview of available evidence related to the implementation and adaptation of health system guidelines evaluated in LMICs. A focus on adaptation and implementation processes is a novel contribution in the literature by examining both of their strategies, interactions and influences. Recognizing the intricacy of contextual factors, we will only be examining implementation and adaptation strategies that directly happened in LMICs. We adopted an integrated knowledge translation approach by collaborating with a broad range of key informants, including the lead of each partner country in the WHO RAISE portfolio, throughout the review process to help ensure that the findings were relevant to knowledge users. Integrated knowledge translation is an approach to research where researchers and end-users work collaboratively to identify relevant knowledge gaps and ensure the production of actionable knowledge [37]. The results of this scoping review provide critical insight into the development of evidence-based implementation and adaptation recommendations for health system guidelines in LMICs.

## Review aims

This scoping review assessed and mapped the available evidence related to adapting and implementing health system guidelines and recommendations in LMICs. The following research questions guided the review:

1. What are the common strategies and approaches for implementing health system guidelines and recommendations in LMICs?
2. What are the common strategies and approaches for adapting health system guidelines and recommendations in LMICs?
3. What are the commonly reported outcomes or indicators of success in adaptation and/or implementation of health system guidelines and recommendations in LMICs?
4. What are the commonly reported barriers and facilitators with respect to adaptation and/or implementation of health system guidelines and recommendations in LMICs?

## Methods

This scoping review was guided by the methodological framework outlined by the JBI [38]. The framework includes six phases: (i) identifying the research question; (ii) searching for studies; (iii) selecting studies; (iv) extracting, charting and appraising data; (v) synthesizing and reporting findings; (vi) consulting with experts and key stakeholders [38].

### Inclusion criteria

#### Population

In alignment with the Effective Practice and Organisation of Care (EPOC) taxonomy of health system interventions [39], this review considered articles including any healthcare organizations, healthcare professionals or healthcare recipients targeted for change by health system guidelines within LMICs.

#### Concept

The concepts relevant for this review consist of the implementation and adaptation strategies, frameworks, and barriers and/or facilitators related to the adaptation and/or implementation of health system guidelines, policies and/or recommendations. Articles were required to explicitly state their intent to implement and/or adapt any evidence-informed health system guideline to be considered for inclusion. Health systems were conceptualized to encompass any system responsible for the provision of health services, finances, and/or governance [40]. Our review considered any evidence-informed (as reported by author) health system guidelines, regardless of the developer. Articles that described their intent to implement and/or adapt clinical practice guidelines were excluded.

Implementation and adaptation, while often undertaken simultaneously, are two distinct concepts being examined by this review. Implementation strategies were defined as any “methods or techniques used to enhance the adaptation, implementation, and sustainability” [14]. Adaptation strategies were defined as a “process of thoughtful and deliberate alteration to the design or delivery of an intervention, with the goal of improving its fit or effectiveness in a given context” [41]. Articles were required to report on the implementation and/or adaptation of health system guidelines to be considered for inclusion.

#### Context

Context in this review involved adaptation and/or implementation strategies applied in LMICs at a health system level. LMICs were defined by the World Bank standards based on gross national income for the 2021 fiscal year [42]. Studies or data related to HICs were excluded from this review.

#### Types of sources

This scoping review considered any quantitative, qualitative or mixed-methods studies that evaluated the implementation and/or adaptation of health system guidelines in any LMICs. Articles that were descriptive in nature (e.g. editorials, commentaries, opinion papers) or did not have evaluation processes for assessing the implementation/adaptation strategy were excluded. Literature reviews that reported on relevant concepts were first reviewed for primary studies and then ultimately excluded. Studies published in English, not restricted by date of publication, were included.

#### Search strategy

The search strategy aimed to locate both published and unpublished studies. An initial search of MEDLINE (Ovid) was undertaken by a librarian scientist to identify relevant studies of interest. The search strategy was developed using Medical Subject Headings (MeSH) terms and keywords contained in the titles and abstracts of relevant articles. A full search strategy for MEDLINE (Ovid) is included in our Additional file 1. This search strategy underwent peer review by another librarian using the Peer Review of Electronic Search Strategies (PRESS) [43] to ensure its accuracy. The search strategy was then adapted for each included information source. Lastly, primary studies from identified literature reviews were scanned for additional studies.

#### Information sources

We employed our search strategy in MEDLINE (Ovid), Embase, CINAHL (Cumulative Index to Nursing and Allied Health Literature), LILACS (Latin American and Caribbean Health Sciences Literature; VHL Regional Portal), and Web of Science databases. Sources of grey literature included a search of the CADTH (Canadian Agency for Drugs and Technologies in Health) Grey Matters Tool, Google, Google Scholar, and ProQuest Dissertations & Theses Global. These databases were chosen to capture potential articles across relevant countries.

#### Study selection

Search results were uploaded into Covidence systematic review software [45] for reference management. To ensure that eligibility criteria were uniformly applied by all reviewers, team members independently pilot-tested 20 citations and met to resolve any areas in need of clarification. Two reviewers then independently screened all titles and abstracts for assessment against the inclusion criteria. Full-text articles of potentially relevant studies were retrieved, and two reviewers independently assessed the full-text studies for eligibility. Disagreements between reviewers were resolved through discussion at each stage of the study selection process. If consensus could not be achieved, a third reviewer made the final decision. Reasons for exclusion of full-text studies were documented and are reported in the Preferred Reporting Items for Systematic Reviews and Meta-Analyses Extension for Scoping Reviews (PRISMA-ScR) flow diagram [46].

#### Data extraction

Data were extracted using a predetermined extraction form to collect key findings relevant to the scoping review questions (Additional file 2). The main concepts in the data extraction form included year of publication, country, study aim(s), study population, setting, funding source, use of theoretical/conceptual frameworks, guideline description, implementation strategies, adaptation strategies, outcomes of interest, study methods, barriers and enablers, key results and stakeholder engagement [38]. Details regarding implementation strategies were extracted based on Proctor and colleagues’ recommendations for operationalizing and reporting implementation techniques [14]. This data extraction framework facilitated the collection of specific and pertinent data related to reported implementation strategies, such as duration, dose and justification. Further, the Framework for Reporting Adaptations and Modifications–Enhanced (FRAME) was used to guide data extraction of adaptation strategies to capture the who, where, when, why and how aspects of modifications [41]. As this review seeks to examine implementation and adaptation as two distinct concepts, data on implementation and adaptation strategies were extracted independently of each other. If articles reported on both implementation and adaptation strategies, concepts related to processes such as barriers, enablers and outcomes were extracted independently. This could only be accomplished if authors explicitly stated which indicators (e.g. barriers, enablers and outcomes) related to which concepts (implementation or adaptation). If this level of detail was not provided, the data were still extracted but we were unable to infer which indicators related to which concepts. Data were also extracted if authors reported using a theoretical/conceptual framework to guide/justify their implementation and/or adaptation techniques. Two reviewers independently extracted details from the included articles, and disagreements were resolved with a third reviewer.

#### Quality assessment

The quality of included studies was assessed using the JBI’s critical appraisal tools and the mixed-methods appraisal tool [47, 48]. Two reviewers independently completed the quality assessment. Any disagreements were resolved through discussion. The results of this quality assessment were not used to exclude studies from the review but rather to provide greater insight into the current body of literature on this topic.

#### Data analysis

We began by categorizing each health system guideline based on the six “building blocks” that WHO identifies as core components to strengthening health systems: (1) service delivery, (2) health workforce, (3) health information systems, (4) access to essential medicines, (5) financing and (6) leadership or governance [49]. Health system guidelines were categorized into these building blocks based on their primary aim. Subsequently, directed content analysis was used to map implementation strategies according to the list of 73 implementation strategies and definitions outlined in the Expert Recommendations for Implementing Change (ERIC) project [28]. The ERIC framework was developed through iterative consultations with experts and literature to derive a comprehensive list of known implementation strategies [28]. Analysis was completed by two reviewers independently, and disagreements were resolved through consensus. Guided by the FRAME, thematic analysis was used to examine and group similarities in adaptation strategies and the who, what, where, why and when of any modification that took place. Lastly, the Capability, Opportunity and Motivation Behaviour (COM-B) model guided the coding of the reported barriers to and enablers of implementation and adaptation [30, 50]. The COM-B model is a theoretically driven, evidence-based framework that outlines a systematic process to identify and understand barriers and enablers with respect to implementation/adaptation of health initiatives [30, 50]. This model also links the identified barriers and enablers to the required mechanisms needed to enact change [51]. Mapping the findings onto published taxonomies, such as the ERIC framework to classify implementation strategies, the FRAME to detail important considerations to adaptation techniques, and the COM-B model to map barriers and enablers, allows for the identification of possible gaps in current knowledge and opportunities for future research [52]. Further, results summaries were stratified per LMIC lending groups (low-, lower-middle and upper-middle-income) and by using WHO’s six building blocks to assess for potential trends [49].

Descriptive summary tables of all included studies were created to outline extracted data specific to the health system guidelines, implementation strategies, adaptation strategies, outcomes/results, and article characteristics. Narrative summaries were included to address each research question.

## Results

**Fig. 1:**
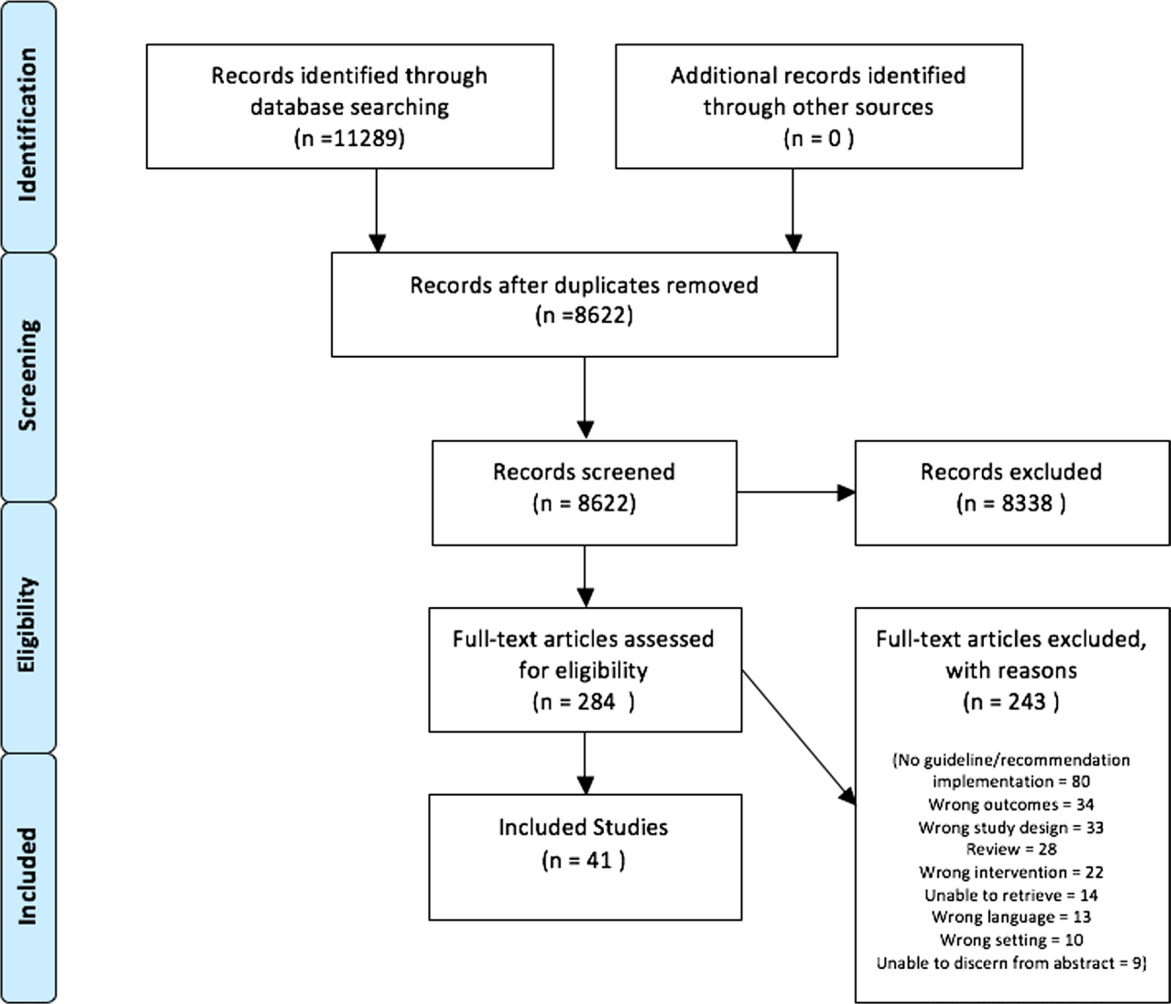
PRISMA diagram

A total of 8622 unique references were identified from the search strategy. No additional citations were uncovered by searching the reference lists of relevant reviews or grey literature sources. After title and abstract screening, 284 papers remained for full-text review. Following this second stage of review, 41 articles were included for data analysis (see Fig. 1 for Preferred Reporting Items for Systematic Reviews and Meta-Analyses [PRISMA] diagram) [53].

### Article summary characteristics

**Fig. 2:**
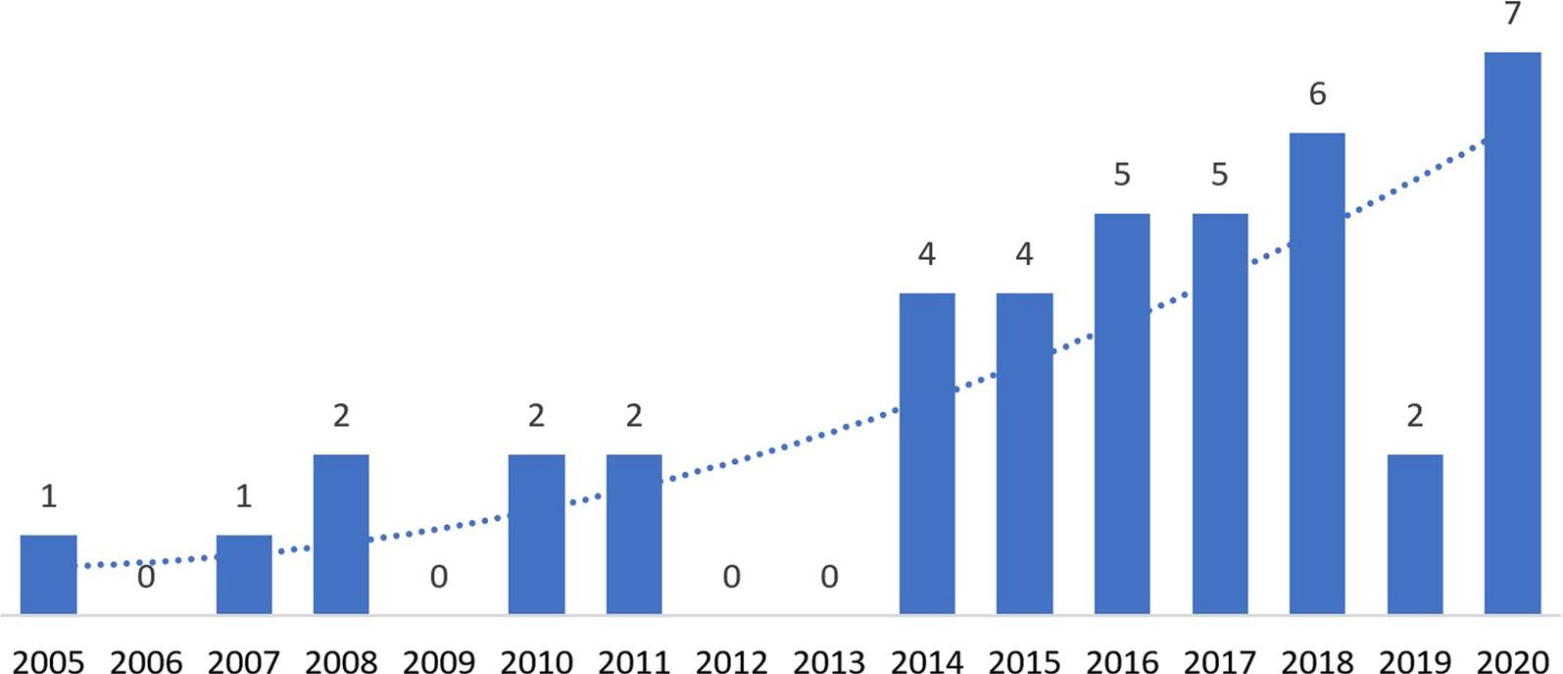
Yearly publication trend

**Fig. 3:**
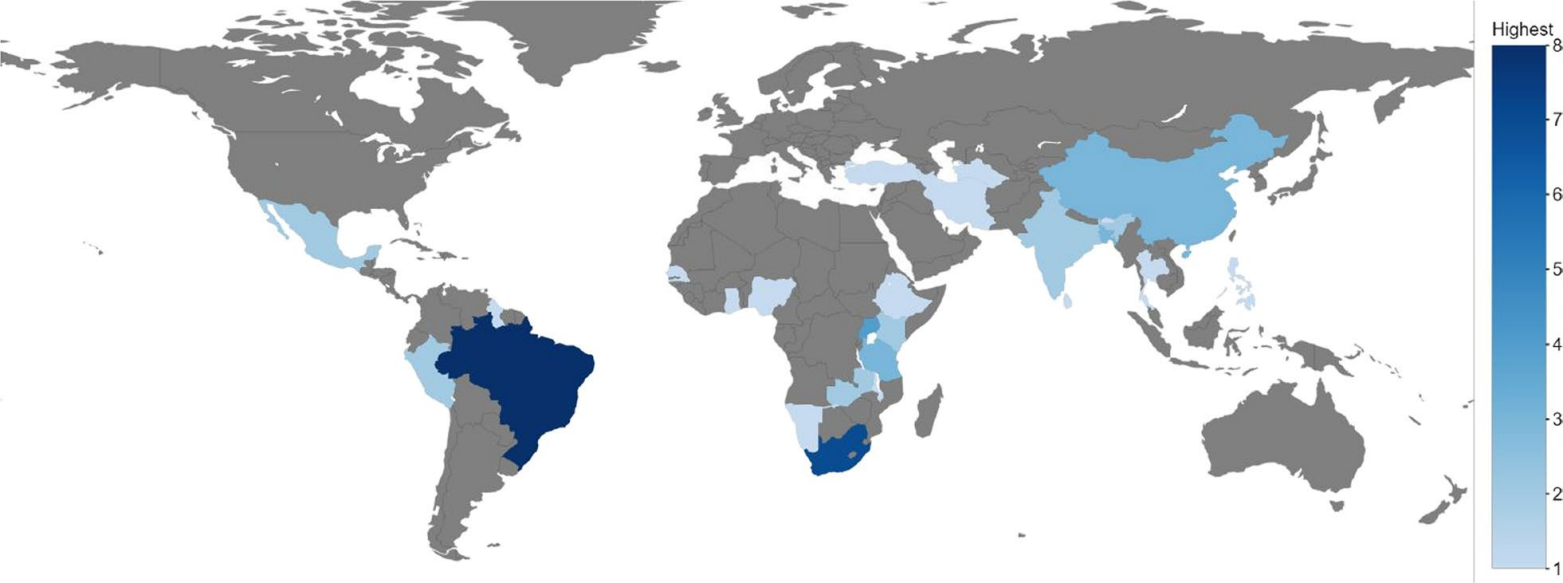
Geographical clustering of health system initiatives

**Fig. 4:**
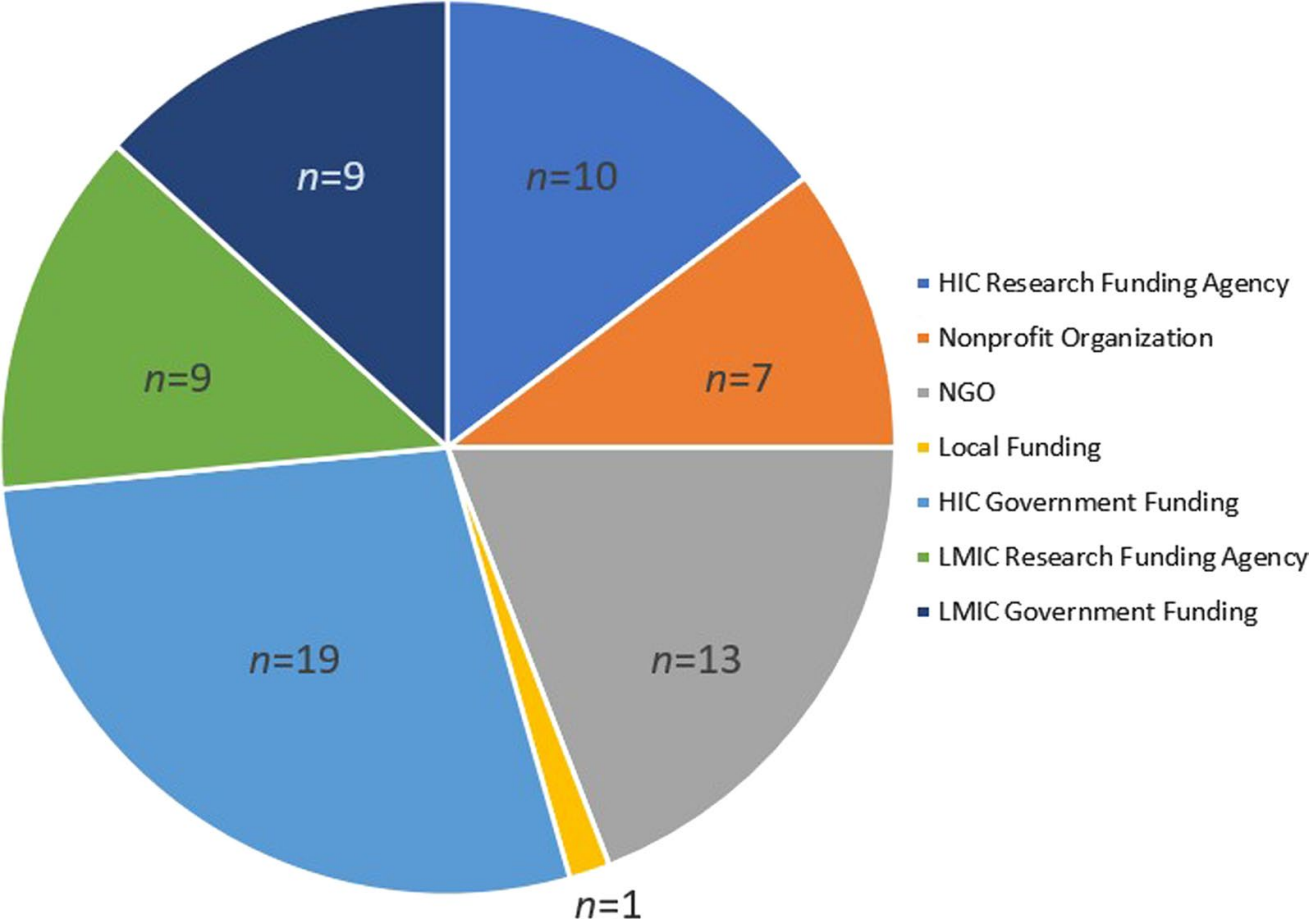
Reported funding sources. *One article may have reported multiple funding sources

Identified articles were published between 2005 and 2010 (*n* = 6), 2011–2015 (*n* = 10), and 2016 and beyond (*n* = 25) (see Fig. 2). Studies were most frequently conducted in upper-middle-income countries (*n* = 21), followed by lower-middle-income countries (*n* = 14) and LICs (*n* = *5*) (see Fig. 3). One study reported on case study findings from low-, middle-, and upper-middle-income countries. Twenty-two studies used qualitative methods, 14 studies employed mixed methods, and five used cross-sectional methods to answer their research questions. Sources of funding varied among studies and often included multiple sources (see Fig. 4). Most studies reported funding from an HIC source (*n* = 21) (e.g. Irish Aid, and United Kingdom’s Wellcome Trust). Other studies reported funding from local country/context initiatives (*n* = 6) and high-income and local country partnerships (*n* = 5). The remaining reported that no funding was received (*n* = 2) or did not report information on funding (*n* = 7). Healthcare workers and end-users were the most commonly targeted study populations. Settings varied across urban and rural locations and community and hospital sites. Articles reported implementing health system guidelines in urban hospitals (*n* = 7), both urban and rural communities (*n* = 7), only urban communities (*n* = 7), and both urban and rural hospitals (*n* = 5). Only one article reported on implementation of a guideline in both urban and rural clinics and hospitals. Please refer to Table 1 for a full summary of article characteristics. Any acronyms used in the tables can also be found in Additional file 3.

**Table 1:** Summary of article characteristics

Year	Author(s)	Country (income bracket)	Funded by	Study methods	Study population	Study setting	Quality appraisal
2008	Amaral et al. [82]	Brazil (upper-middle-income)	Bill & Melinda Gates Foundation	Cross-sectional ecological study	Healthcare professionals, health system organization, family and community practice	Municipalities with a population between 5000 and 50,000 inhabitants	100% (high)
2011	Blanco-Mancilla [84]	Mexico (upper-middle-income)	Not reported	Qualitative	Medical professionals who interact with service users or patients	Hospitals and health centres	100% (high)
2007	Leethongdee [83]	Thailand (upper-middle-income)	Royal Thai Government Office of Educational Affairs (Kor-Por London) Civil Service Commission Office (Kor-Por Thailand)	Qualitative	Personnel who worked in the public healthcare system overseen by the ministry of health	Public health	100% (high)
2018	Zakumumpa et al. [85]	Uganda (low-income)	Consortium for Advanced Research Training in Africa (CARTA) Wellcome Trust (United Kingdom) Department for International Development (DFID) Carnegie Corporation of New York Ford Foundation MacArthur Foundation	Mixed-methods sequential explanatory	Heads of the ART clinic, head nurses, HR managers, clinicians, finance managers, strategy directors	Various health facilities in peri-urban settings or urbanized parts of rural areas	100% (high)
2020	Miguel-Esponda et al. [69]	Mexico (upper-middle-income)	No financial support received	Mixed-methods convergent study design	Service users registered in the health information system (HIS)	Ten rural primary healthcare (PHC) clinics supported by CES [Compañeros En Salud]	93% (high)
2020	Callaghan-Koru et al. [86]	Bangladesh (lower-middle-income)	United States Agency for International Development (USAID)	Qualitative case study	Mothers with children giving birth	In hospital setting—birthing units	90% (high)
2020	Mutabazi et al. [87]	Sub-Saharan Africa (low-income)	Canadian Institute of Health Research (CIHR) (Canada) Integrated Intervention for Diabetes Risk after Gestational Diabetes in South Africa (IINDIAGO) (South Africa)	Descriptive qualitative study	Pregnant women, women in labour/delivery and breastfeeding, frontline workers	Public health facilities	90% (high)
2018	Saddi et al. [88]	Brazil (upper-middle-income)	Graduate Studies Coordination Board (Coordination for the Improvement of Higher Education Personnel [CAPES]) Brazilian Ministry of Education Federal University of Goiás (UFG) Office of the Dean of Extension and Research	Contingent mixed-methods approach	Frontline health workers; managers, nurses	Healthcare units in Goiânia; primary care setting	86% (high)
2015	Xia et al. [89]	China (upper-middle-income)	Centre for Environment and Population Health (Griffth University)	Mixed methods	Pregnant women service users	Maternal and child healthcare hospitals	86% (high)
2014	Armstrong et al. [90]	Tanzania (lower-middle-income)	Evidence for Action Tanzania	Qualitative	Healthcare professionals, health system coordinators, district, region and zonal health administrators	One regional referral hospital, one government district hospital and one faith-based district hospital	80% (high)
2011	Ditlopo et al. [91]	South Africa (upper-middle-income)	Irish Aid	Qualitative case study design	Policy-makers, hospital managers, nurses and doctors	Predominantly district rural hospitals	80% (high)
2017	Doherty et al. [92]	Uganda (low-income)	Swedish and Norwegian government agencies South African Medical Research Council	Descriptive qualitative	Implementation partners, Ministry of Health, multilateral agencies (UNICEF and WHO), district management, community- and facility-based health workers	All four regions of the country	80% (high)
2019	Lovero et al. [93]	South Africa (upper-middle-income)	National Institute of Mental Health (NIMH) Wainberg/Arbuckle Training Grant United States President’s Emergency Plan for AIDS Relief (PEPFAR)	Mixed-methods exploratory design	District-level programme managers (DPMs)	Urban and rural primary care clinics throughout district	80% (high)
2014	Mkoka et al. [94]	Tanzania (lower-middle-income)	Swedish International Development Cooperation Agency (Sida)	Qualitative approach	District medical officer (DMO), district nursing officer (DNO), district health officer (DHO), district health secretary (DHS), and district pharmacist (DP)	A typical rural district	80% (high)
2016	Moshiri et al. [95]	Iran (upper-middle-income)	School of Public Health Research Deputy of the Tehran University of Medical Sciences (TUMS)	Qualitative	Designers of public health facilities, provincial health managers, community health workers and two former health ministers	Rural healthcare facilities	80% (high)
2020	Muthathi et al. [96]	South Africa (upper-middle-income)	South African Research Chairs Initiative (SARChI) Department of Science and Innovation (South Africa) National Research Foundation (South Africa) Atlantic Philanthropies	Nested qualitative study	Health policy actors: national government, provincial government head office, district, subdistrict and local government	Urban and rural provinces	80% (high)
2017	Schneider and Nxumalo [97]	South Africa (upper-middle-income)	Canadian International Development Research Centre (IDRC) Funded through a variety of other mechanisms that were not reported	Qualitative case study	Community health	Community care, primary care clinics	80% (high)
2010	Sheikh et al. [98]	India (lower-middle-income)	Aga Khan Foundation’s International Scholarship Programme DFID TARGETS Consortium at the London School of Hygiene & Tropical Medicine (LSHTM) University of London Central Research Fund	Qualitative case study	Public health authorities, hospital administrators, medical practitioners	Public health facilities Private health	80% (high)
2016	Shelley et al. [99]	East Africa (lower-middle-income)	DFID (United Kingdom)	Qualitative approach	Healthcare workers	Rural community healthcare	80% (high)
2019	Zhou et al. [67]	China (upper-middle-income)	China Medical Board China Postdoctoral Science Foundation Central South University Post-Doctoral Science Foundation	Mixed methods	Senior leaders, department directors from a town hospital, family members of patients	Liuyang Mental Health Prevention and Treatment Center (MHC)	80% (high)
2018	Carneiro et al. [100]	Brazil (upper-middle-income)	Not reported	Cross-sectional quantitative descriptive	Physicians	Isolated primary care facilities in Marajó	75% (high)
2014	Costa et al. [101]	Brazil (upper-middle-income)	No financial support received	Cross-sectional evaluative quantitative study	Doctors completing home visits and nurses providing individual care	Municipalities within Brazil	75% (high)
2018	Sami et al. [102]	South Sudan, Africa (low-income)	Save the Children’s Saving Newborn Lives programme ELMA Relief Foundation	Mixed-methods case study	Newborns and mothers	Community/facility-based settings including PHC centre, community health programme centres, hospital and camps	73% (high)
2015	Febir eta al. [103]	Ghana (lower-middle-income)	Bill & Melinda Gates Foundation ACT [artemisinin-based combination treatment] Consortium	Qualitative study	Healthcare workers	District hospital, health centres and community-based health services	70% (high)
2017	Pyone et al. [104]	Kenya (lower-middle-income)	DFID UKAid	Qualitative methods	10 national-level policy-makers, 10 county health officials and 19 healthcare providers	10 district- and county-level hospitals and other health facilities in selected counties	70% (high)
2020	Rahman et al. [105]	Bangladesh (lower-middle-income)	GlaxoSmithKline (GSK) through PATH (Seattle, USA)	Qualitative descriptive	Key stakeholders, health service providers and caregivers	At both the national and district levels of Khulna and Lakshmipur, specifically in two subdistrict public healthcare facilities	70% (high)
2008	Stein et al. [106]	South Africa (upper-middle-income)	IDRC (Canada)	Qualitative methods	PHC nurses	Urban and rural PHC settings	70% (high)
2017	Bergerot et al. [79]	Brazil (upper-middle-income)	Not reported	Mixed methods	Psychologists and oncology staff; patients aged 18 or older, with cancer treatment plan	Hospitals and cancer centres from different Brazilian cities	66.66% (medium)
2010	Halpern et al. [77]	Guyana (upper-middle-income)	Not reported	Cross-sectional	Doctors, nurses and data entry clerks from each care and treatment site	Clinics across the nation	62.50% (medium)
2020	Ejeta et al. [107]	Ethiopia (low-income)	Not reported	Qualitative descriptive	Three hospitals in Ethiopia Families within	The health facility sites located in Addis Ababa, Bishoftu and Hawassa	60% (medium)
2016	Smith Gueye et al. [108]	Bhutan, Mauritius, Namibia, Philippines, Sri Lanka, Turkey and Turkmenistan (low-, middle- and upper-middle-income)	Bill & Melinda Gates Foundation Malaria Elimination Initiative of the Global Health Group (USA)	Qualitative case study review	Healthcare and programme staff	Mostly in decentralized health systems	60% (medium)
2020	Ryan et al. [109]	Nigeria (lower-middle-income)	CBM Consultancy (Australian Government department) Comprehensive Community Mental Health Programme (CCMHP)’s monitoring and evaluation budget	Mixed-methods manualized case study	Project coordinator, community mental health project officer, self-help group, development project officer and six community psychiatric nurses	Urban and semi-urban mental health clinics (some rural)	60% (medium)
2017	Andrade et al. [75]	Brazil (upper-middle-income)	Not reported	Cross-sectional observational case study	Pregnant women or women with children under 2, suffering from chronic conditions and/or diabetes and hypertension	Primary and secondary healthcare	50% (medium)
2014	Roman et al. [66]	Africa (lower-middle-income)	USAID	Qualitative observational case study	Pregnant women in Africa	Health system area	50% (medium)
2016	Investigators of WHO Low Birth Weight (LBW) Feeding Study Group [110]	India (lower-middle-income)	WHO (Geneva)	Mixed-methods before-and-after study	Healthcare practitioners and parents of LBW babies	First-referral-level health facilities	33% (low)
2016	Lavôr et al. [111]	Brazil (upper-middle-income)	Not reported	Mixed-methods multiple-case study	Nurses	Basic health units and four outpatient clinics, called specialty polyclinics	27% (low)
2005	Bryce et al. [58]	Bangladesh, Brazil, Peru, Tanzania, Uganda (lower-middle-income)	Bill & Melinda Gates Foundation USAID	Mixed methods	Health facilities with or without integrated management of childhood illness	Health facilities	20% (low)
2018	Kihembo et al. [57]	Uganda (lower-middle-income)	DFID WHO-AFRO Continuum of Care for Reproductive, Maternal, Newborn, Adolescent and Child Health (RMANCH) USAID UNICEF Global Polio Eradication Initiative United Nations Central Emergency Response Fund (CERF) WHO (Uganda)	Qualitative descriptive study	Health workforce	District- and regional-level referral hospitals	20% (low)
2015	Li et al. [112]	China (upper-middle-income)	Law Department of National Health and Family Planning Committee Jinan Science & Technology Planning Project	Mixed-methods field observation	Personnel of the health department of Shandong Province and health departments, directors, medical personnel of township hospitals	Six township hospitals and three village clinics	6.60% (low)
2015	Wingfield et al. [113]	Peru (upper-middle-income)	Wellcome Trust Innovation for Health and Development (FHAD) and the Joint Global Health Trials Consortium of the Wellcome Trust United Kingdom Medical Research Council DFID Bill & Melinda Gates Foundation British Infection Association Imperial College Centre for Global Health Research	Mixed methods	Project team, project participants, civil society and stakeholders	Two suburbs of Peru’s capital, Lima	6.60% (low)
2018	Kavle et al. [114]	Kenya (lower-middle-income)	USAID	Qualitative	Mothers	Community care health facilities	0% (low)

### Health system guidelines

Table 2 summarizes the health system guidelines implemented in the included studies. While specific guidelines varied across studies, out of the total 41 studies, three reported on implementation of the Integrated Management of Childhood Illness (IMCI) guidelines and another three outlined the Prevention of Mother-to-Child Transmission of HIV/AIDS guidelines.

**Table 2:** Health system guideline/recommendation overview

Author/year	Guideline/recommendation name	Study aim and objectives	Description	Health system building block
Amaral et al. (2008) [82]	Integrated management of childhood illnesses (IMCI)	Describe factors associated with the implementation of IMCI in north-eastern Brazil	IMCI aims to reduce mortality and morbidity associated with childhood diseases by improving three key components: (1) performance of health professionals using standardized protocols; (2) improving the health system organization by means of adequate support for the availability of resources; (3) health promotion practices through family and community-based activities	Service delivery
Andrade et al. (2017) [75]	Attention to chronic conditions model (ACCM) was adapted to create lab for innovations in chronic conditions (LIACC)	Address implementation of LIACC Document the main challenges and lessons learned to suggest a more suitable chronic care model at the municipal level	Adapted from the seven steps of ACCM, LIACC implements four macro processes used as a management tool in primary healthcare (PHC) for chronic conditions: (1) evaluation of infrastructure; (2) focus on primary care to acute health services; (3) management and monitoring of chronic conditions; (4) management and monitoring of home healthcare visits	Service delivery
Armstrong et al. (2014) [90]	Maternal and perinatal death reviews (MPDR)	Explore the current implementation of MPDRs in Tanzania	MPDR encourages multidisciplinary team discussions from staff involved in the patients’ care as well as a review of the patients’ documentation to identify avoidable factors and opportunities for improvement	Health workforce
Bergerot et al. (2017) [79]	Psycho-oncology programme	Characterize the use of screening measures for psychologists from different oncology services Present the preliminary results from this programme implementation and development	The programme was subdivided into six actions: screening of distress, anxiety, depression, quality of life; classification of risk criteria; discussion by the psychology team; synthesis and discussion with healthcare team; evidence-based results analysis; treatment plan and record in medical records	Service delivery
Blanco-Mancilla (2011) [84]	Popular health insurance (PHI) programme	Understand why health policies differ across Mexico City Identify issues that contribute to the success or failure of translating policy into practice	Providing healthcare coverage to previously excluded populations	Service delivery
Bryce et al. (2005) [58]	MCI strategy	Compare the programme (IMCI) expectation findings of the Multi-Country Evaluation of IMCI Effectiveness, Cost and Impact (MCE-IMCI) to the five most important programme expectations from the IMCI impact model	IMCI is a strategy for reducing mortality among children under the age of 5 years UNICEF, WHO and their technical partners developed the strategy in a stepwise fashion, seeking to address limitations identified through experience with disease-specific child health programmes, and those addressing diarrhoeal disease and acute respiratory infections	Service delivery
Callaghan-Koru et al. (2020) [86]	Chlorhexidine (CHX) cleansing policy	Identify and compare facilitators of and barriers to the institutionalization and expansion strategies of the national scale-up of CHX	Prioritizes several newborn health interventions such as kangaroo mother care, management of newborn infections and ensuring essential newborn care including the application of CHX to the umbilical cord	Service delivery
Carneiro et al. (2018) [100]	More physicians for Brazil programme (MPBP) as part of the Family Health Strategy (FHS)	To evaluate the performance of the FHS, through the deployment of MPBP in Marajó-Pa-Brazil	Broadening the access to basic healthcare services and connecting the teams to individuals, families and communities in the complex task of taking care of life	Access to essential medicine
Costa et al. (2014) [101]	FHS	To re-evaluate the implementation of the FHS in the state of Santa Catarina between 2004 and 2008 by considering indicators of potential coverage, evidence of change in the care model, and the impact on hospitalizations	Characteristics of the FHS are teamwork and ascribed distribution of patients, with a forecasted number of families/individuals under its responsibility Proactive approach to the health of the community ascribed which relies on territorialization, family registers, diagnoses of health situations and health initiatives developed in partnership with the community	Service delivery
Ditlopo et al. (2011) [91]	Rural allowance policy	Analyse policy implementation and effectiveness and its influence on motivation and retention	Attract and retain health professionals to work full-time in public health services in rural, underserved and other inhospitable areas identified by provincial health departments	Financing
Doherty et al. (2017) [92]	Prevention of mother-to-child transmission of HIV/AIDS (PMTCT) (Option B+)	Present findings from a rapid assessment of PMTCT Option B+ implementation in Uganda 3 years after policy adoption	PMTCT evolved progressively from single-dose nevirapine prophylaxis in 2000 to the current recommendation that all pregnant and breastfeeding women, irrespective of CD4 count, should receive lifelong antiretroviral treatment (ART), known as Option B+	Service delivery
Ejeta et al. (2020) [107]	Strengthening Ethiopia’s Urban Health Promotion (SEUHP) implemented the Urban Community Health Information System (UCHIS)	Document the challenges and lessons learned in the UCHIS implementation process	Each of the 15 health service packages identified contained service cards and tally sheets to help improve data collection and standardization	Health information system
Febir et al. (2015) [103]	Integration of rapid diagnostic test (RDT) in IMCI	Evaluate and report the issues health workers faced in integrating RDT management into their working practices	In 2010 IMCI was adapted wherein case management of malaria should be a test-based approach, and therefore the integration of a rapid diagnostic test (RDT)-based intervention was undertaken	Service delivery
Gueye et al. (2016) [108]	Malaria elimination programmes: Global technical strategy for malaria (GTS) Action and Investment to defeat Malaria (AIM) Global Malaria Eradication Programme (GMEP)	Examine countries in different socioeconomic, political and ecological contexts and evaluate how the health system has operated within the context of different political, financial and human resources activities Identify how countries have implemented elimination programmes, and adapted their malaria elimination strategies	GTS: provided the framework for achievement of elimination and establishing an elimination goal for 35 countries. Programme to reach global goals for malaria control, elimination and eventually eradication AIM: an action framework to reduce malaria through the Roll Back Malaria Partnership GMEP: based on vertical time-limited interventions deployed through centralized health systems at the national level	Service delivery
Halpern et al. (2010) [77]	The patient monitoring system (PMS) for patients with HIV	Describe the process used to implement PMS Provide examples of the programme-level data Highlight benefits for national programmes	PMS is used for patient care and data collection The physical components of the WHO HIV care and ART PMS include a patient chart, two patient registers, and cross-sectional and cohort analysis reporting form	Health information system
Investigators of WHO Low Birth Weight (LBW) Feeding Study Group (2016) [110]	LBW feeding guidelines in first-referral-level health facilities	Evaluate the effect of implementing WHO LBW feeding guidelines	Guidelines aim to improve knowledge and skills of health workers Guidelines for optimal feeding of LBW infants, to improve care and survival of LBW infants	Health workforce
Kavle et al. (2018) [114]	Baby-Friendly Community Initiative (BFCI)	Describe the implementation process Discuss success, challenges, lessons learned and opportunities for integration into other health areas	Through mother-to-mother community support groups, BFCI addresses breastfeeding and nutrition challenges by providing educational interventions in community gardens, water, sanitation and hygiene	Service delivery
Kihembo et al. (2018) [57]	Integrated Disease Surveillance and Response (IDSR)	Describe the design and process of IDSR revitalization, highlighting the rollout of the revised IDSR guidelines through structured training of the health workforce up to the operational level nationwide	Strategy aimed at strengthening integrated, action-oriented public health surveillance and response at all levels of the health system Focused on detection, registration, conformation, reporting, data analysis and provision of feedback	Service delivery
Lavôr et al. (2016) [111]	Directly observed treatment, short-course (DOTS)	Assess the degree of implementation of the DOTS strategy for tuberculosis (TB) in a large city	DOTS is based on five fundamental components: sustained political and financial commitment; diagnosis through quality-ensured sputum-smear microscopy; standardized short-course anti-TB treatment; a management system for uninterrupted supply of anti-TB drugs; information system that allows monitoring and evaluation of actions and their impacts	Access to essential medicine
Leethongdee (2007) [83]	Universal coverage (UC) healthcare reform	Understand the factors influencing the implementation at a local level Build a general account of the reforms that fit each of three individual provincial cases	UC reform objective was to reduce geographical inequalities in funding and workflow distribution, problems in resource allocation, lack of progress in developing primary care, and tension between curative and preventative care approaches	Financing
Li et al. (2015) [112]	WHO essential drugs policy	Analyse the impact on village-level and township-level health service system Summarize the effectiveness of implementing essential drugs policy; identify the problems of various aspects Conduct an in-depth analysis of the causes, and provide ways to improve the essential drugs policy	Essential drug policy aims to improve the availability of essential drugs and to promote rational drug use	Access to essential medicine
Lovero et al. (2019) [93]	The National Mental Health Policy Framework and Strategic Plan 2013–2020 (the Strategic Plan)	Gain knowledge on stepped-care procedures for management of mental illness in primary care services Determine the degree to which integrated procedures have been implemented Identify challenges encountered in coordination of integration efforts	The Strategic Plan aims to fully integrate mental health assessment and management services, including screening, management of mental disorders, referral pathways and training, into all aspects of primary care, with an emphasis on TB, HIV and antenatal care services The strategic plan was to be coordinated at the district administrative level	Service delivery
Miguel-Esponda et al. (2020) [69]	Compañeros En Salud (CES) mental health programme	Assess the implementation of the CES programme to understand the extent of success in integrating mental health into PHC Determine strengths and limitations of the success or failure of integration To determine managers’ and providers’ perspectives on the programme Determine the key strengths and remaining challenges to the implementation of the CES mental health programme	CES aims to strengthen the PHC system to improve access to quality healthcare The organization facilitates the delivery of general health services (including mental health) in 10 PHC clinics. For mental health, a coordinator oversees the delivery of mental health services and capacity-building activities and provides support for the management of complex cases All mental health services are delivered by medical doctors (MDs) Services are designed according to adapted clinical guidelines and include case identification, diagnosis, pharmacological treatments, individual and group talk-based interventions, and home visits	Service delivery
Mkoka et al. (2014) [94]	Emergency obstetric care (EmOC)	Explore the experiences and perceptions of a council health management team (CHMT) in working with multiple partners while illuminating some governance aspects that affect implementation of EmOC at the district level	Strategy aims to strengthen all dispensaries and health centres through provision of basic EmOC (BEmOC) by strengthening the capacity of district hospital and upgrade by 50% health centres to provide comprehensive EmOC and strengthening health workers competencies	Service delivery
Moshiri et al. (2016) [95]	PHC	Investigation of context, content, actors and process of PHC implementation Investigation of the referral system situation in Iran from 1982 to 1989	In order to tackle physician shortages, foreign doctors were being hired en masse to support PHS implementation	Service delivery
Mutabazi et al. (2020) [87]	PMTCT	Explore the perspective of experts and other key informants on the PMTCT integration into PHC	Strategy involving the integration of testing to reduce mother-to-child transmission during different phases of pregnancy	Service delivery
Muthathi et al. (2020) [96]	Ideal clinic realization and maintenance (ICRM) programme	Generate knowledge on the policy implementation Examine the influence of motivation, cognition and perceived power of the policy actors and how it influenced ICRM implementation Explore policy coherence in the ICRM programme Explore the perceptions of stakeholders at the national, provincial and local government levels	The goal of the ICRM programme is to prepare all PHC facilities to meet the quality standards set by the Office of Health Standards Compliance (OHSC) An ideal clinic is defined as a clinic with good infrastructure, adequate staff, adequate medicines and supplies, and good administrative processes, with sufficient bulk supplies; it uses applicable clinical policies, protocols and guidelines, and it harnesses partner and stakeholder support	Health workforce
Pyone et al. (2017) [104]	Free maternity services (FMS) policy	Understand how the policy changed health system governance in Kenya and use the insights to inform policy implementation in Kenya and in other LMICs	FMS was part of a national strategy to reduce maternal and neonatal mortality, alleviate poverty and achieve the Millennium Development Goal targets; abolish user fees for all health services and dispensaries, and provide FMS in all levels of care of the government health sector	Financing
Rahman et al. (2020) [105]	Maternal, neonatal, child and adolescent health (MNC&AH) and community-based healthcare (CBHC), reproductive and adolescent health (MCR&AH)	Understand key drivers for implementation of WHO recommendations for the case management of childhood pneumonia and possible serious bacterial infection (PSBI) with amoxicillin dispersible tablets (DT) Generate evidence to strengthen newborn and child health programmes in Bangladesh	The Ministry of Health and Family Welfare (MOHFW) in Bangladesh provides healthcare services for childhood pneumonia and PSBI in the PHC setting through both the directorate of health services and directorate of family planning, under three operational plans Incorporate child-friendly amoxicillin DT for the case management of childhood pneumonia and PSBI when referral for oral amoxicillin is not feasible	Service delivery
Roman et al. (2014) [66]	Malaria in pregnancy (MIP)	Assess how three countries in Africa were able to achieve greater progress in MIP control Identify the practices and strategies that supported the success of the MIP programme Identify bottlenecks in MIP programme implementation processes Share lessons learned	The MIP framework aims to prevent and control malaria during pregnancy by focusing on three methods that stabilize transmission: (1) intermittent preventative treatment with sulfadoxine/pyrimethamine (SP) antimalarial drug; (2) use of physical insecticide nets; (3) effective case management based on signs and symptoms	Service delivery
Ryan et al. (2020) [109]	Comprehensive community mental health programme (CCMHP)	Aims to help inform the utilization of public–private partnerships (PPPs) for mental health policy implementation in Nigeria and other low-resource settings by documenting a promising example from Benue	Two community-based rehabilitation facilities operate under CCMHP CCMHP procures medicines from CHAN Medi-Pharm and sets up Drug Revolving Fund at each health centre to ensure constant supply Referrals are made directly between the community psychiatric nurse (CPN) or community health extension worker (CHEW) and specialists at Federal Medical Centre Makurdi or Benue State University Teaching Hospital CPNs receive formal training, retraining and accreditation, funded by CCMHP CCMHP trains people as community-level mental health advocates for promotion, identification and referral. CPNs and CHEWs conduct community outreach for follow-up	Service delivery
Saddi et al. (2018) [88]	Brazilian national programme for improving primary care access and quality (PMAQ)	To determine frontline worker adherence to PMAQ and their perception of the impact of the programme Determine the relationship between the impact of the PMAQ as perceived by frontline workers and the way they evaluate the organizational capacity of the FHS at the front line	This programme was adopted in 2011 to improve the quality and performance of PHC in Brazil, which is broadly known through its main policy: the FHS PMAQ objectives are (1) to promote quality and innovation in primary care management, strengthening self-assessment, monitoring and assessment, institutional support and permanent education processes; (2) to improve the use of information systems as a primary care management tool; (3) to institutionalize a primary care assessment and management culture; (4) to stimulate the focus of primary care on the service user, promoting management processes and transparency	Service delivery
Sami et al. (2018) [102]	WHO standards for community- and health facility-based newborn care	Examines the feasibility of implementing a package of community- and facility-based neonatal interventions	WHO standards for community- and health facility-based newborn care prioritized the most critical services (neonatal interventions for reducing mortality) during a humanitarian crisis	Service delivery
Schneider and Nxumalo (2017) [97]	Ward-based outreach team (WBOT) strategy—adaptation for community health worker programme	Understand the leadership and governance structure Assess the provincial experiences with adoption and implementation of the WBOT strategy	Established set of proposals for the reorganization of community-based services	Leadership/governance
Sheikh et al. (2010) [98]	HIV testing policies	Investigate problems in the implementation of standardized public health practice guidelines from the perspective of the participant actors	Focused on the following aspects of the policy: (1) informed consent; (2) HIV testing as a precondition to preforming a medical procedure; (3) strict confidentiality	Health workforce
Shelley et al. (2016) [99]	National community health worker (NCHW) strategy	Evaluate implementation process Determine barriers and facilitators Assess how evidence was used to guide ongoing implementation and scale-up decisions	A strategy developed to recruit community health assistants for assistance with disease burden through a comprehensive PHC curriculum Strategy aimed to reduce maternal and child mortality by providing PHC services as close to the family as possible	Health workforce
Stein et al. (2008) [106]	Practical Approach to Lung Health in South Africa (PALSA) PLUS programme	Explore the value of PALSA PLUS guideline training approach from a PHC nurse perspective Evaluate the strategies used for adoption	Health system-based approach to training for primary care providers with two components: (1) a comprehensive set of algorithm-based syndromic guidelines for PHC nurse clinical management of respiratory disease and HIV/AIDS; (2) a training programme to facilitate guideline implementation	Service delivery
Wingfield et al. (2015) [113]	CRESIPT: community randomized evaluation of a socioeconomic intervention to prevent TB	Evaluate a socioeconomic intervention to support prevention and cure of TB in TB-affected households Describe the challenges of implementation, lessons learned and refinement of TB intervention	The CRESIPT project aimed to evaluate a socioeconomic intervention (via cash transfers) to support prevention and cure of TB in TB-affected households and, ultimately, improve community TB control	Financing
Xia et al. (2015) [89]	PMTCT; prenatal HIV, syphilis and hepatitis B testing (PHSHT)	Examine the challenges and effectiveness of integrating PHSHT services	A priority strategy (promoted by WHO) involving the integration of services including testing to reduce mother-to-child transmission (MTCT)	Service delivery
Zakumumpa et al. [85]	ART scale-up	Explore how different health system components interact in influencing the sustainability of ART scale-up implementation	Provision of free antiretroviral drugs, workforce training in ART management, enhancing laboratory capacity and strengthening ART programme reporting	Access to essential medicine
Zhou et al. (2019) [67]	The mid- and long-term policy and development plan for mental health in Liuyang Municipality (Liuyang policy and Liuyang plan)	Address the gap in China’s mental health policy literature with respect to local-level promotion and implementation Provide a deeper understanding of China’s problems and general lessons for implementing mental health policy at the local level	The four main objectives of Liuyang policy and Liuyang plan include (1) establishing a leadership and coordination mechanism for mental health work; (2) constructing a three-level network of mental health services; (3) management and intervention for patients with psychosis (PWP); and (4) improving the public’s awareness and knowledge of mental health	Leadership/governance

Service delivery was the health system building block most frequently targeted by the identified guidelines (*n* = 24). The remaining building blocks were targeted as follows, in descending order: health workforce (*n* = 5), financing (*n* = 4), access to essential medicine (*n* = 4), health information system (*n* = 2), and leadership and governance (*n* = 2).

### Adaptation strategies

Only 14 articles explicitly reported on the concept of adaptation. Rarely did articles specifically comment on the strategies used to determine what and why adaptations were necessary. Those that reported how adaptations occurred often described any modifications as being suggested solutions to identified challenges during both pre- and post-implementation. Three articles also described a dedicated multidisciplinary working group aimed to gather feedback and identify required modifications. Six articles reported adaptations to be reactive in nature and another six reported them to be proactively planned. Modifications made were frequently reported as adding, tailoring or tweaking content elements, such as the addition of training sessions, expanding scope of practices and restructuring funding sources. None of the included articles reported using a guiding framework to help identify areas where adaptation could be beneficial and/or necessary. A full summary of the adaptation strategies and their related concepts according to the FRAME is given in Table 3.

**Table 3:** Health system guideline/recommendation adaptation strategies (FRAME)

Author	Adaptation strategies	Justification	When the modification occurred; was adaptation planned	Who participated in the decision to modify	What was modified; content of modification	Level of delivery
Andrade et al. (2017) [75]	Attention to chronic conditions model (ACCM)	Lack of resources	Not reported; planned proactive	Steps were conditioned for the ability of health professionals to understand the seven macro processes and their engagement based on available resources	The seven steps of the ACCM (they cut three of the steps to adapt to this health system); removing/skipping elements	Health professionals are the primary and secondary level of care
Armstrong et al. (2014) [90]	Maternal and perinatal death reviews (MPDR) system implementation	Adaptations based on challenges that were identified through a case review including lack of training	These are suggested solutions to challenges that were identified—may or may not have been put into practice; reactive	Determined these during an MPDR meeting	Training and evaluation—providing skills and education to maternity staff and women in the community, respectively; adding elements—training and education	Community (women) and clinic/unit level (maternity staff at hospital/reproductive and child health coordinator)
Bryce et al. (2005) [58]	IMCI generic guidelines can be adapted by any country or area to reflect their specific epidemiological profile and health system characteristics WHO worked to develop guidelines for the country adaptation process, including evidence for intervention choices, models for how to incorporate additional diseases and conditions into the training materials, and how to conduct local studies to identify terminology and local foods Cadres of “IMCI adaptation consultants” were trained at regional and global levels	Review of the guideline expectations	Pre-implementation and early implementation; proactive	Countries that implement this programme adapt it to fit their local context	Contextual—setting; tailoring to their local context	Target intervention group
Carneiro et al. (2018) [100]	The more physicians in Brazil programme (MPBP) has resulted in changes in the work processes of the Family Health Strategy (FHS), including changes to the management and control models used in the region	Municipalities experienced strong ascending trends in the number of prenatal consultations and lack of access to resources	Implementation; reactive	Ministry of Health (MoH)	Contextual—how treatment is delivered; tailoring/tweaking/refining—reorganization of the prenatal care	Target intervention group
Gueye et al. (2016) [108]	Strategies were adapted to implement management of malaria programme Introducing new or adapting strategies, from insecticide rotation to lessen the risk of insecticide resistance, to an increase in parasitological screening in development areas to curtail the risk of transmission, to collaborations with the private sector	None reported	Early implementation; reactive	Staff	Contextual; tailoring to local context	Organization
Halpern et al. (2010) [77]	Adaptation of a standardized HIV patient monitoring system (PMS) WHO provided training on the HIV care and antiretroviral treatment (ART) PMS, and the technical working group adapted each component for Guyana System tools and functions were modified based on feedback from the training session participants, and a pilot PMS was subsequently implemented at one site	None reported	Pre-implementation; planned/proactive	Technical working group	Contextual—patient chart data elements and functionality to PMS system; tailoring/tweaking, adding elements to patient chart	Clinic-unit level—HIV care ART
Kihembo et al. (2018) [57]	Implement nationwide ISDR training to health facilities based on the revised guidelines developed Post-training support through integrated supervision	Two challenges from the first implementation: Lack of funding resulted in a lack of resources and capacities at the operational level A need for a harmonized outbreak response and information flow at the district level	Pre-implementation; planned	Ministry of health along with key partners	Aimed to enhance the capacity of districts to promptly detect, access and effectively respond to public health emergencies; adding elements—training	Health workforce all the way up to the operational national level
Leethongdee (2007) [83]	Government decided to fund the scheme by pooling the Ministry of Public Health (MoPH) budgets for public hospitals, other health facilities, and Medical Welfare Scheme (MWS) and voluntary health card scheme and providing additional money	The initial plan met resistance from quarters such as the civil service and the labour unions	Pre-implementation; reactive	Civil service and labour unions rejected the initial plan, government then had to reassess	Implementation and scale-up activities; substituting the funding structures	Target intervention group
Mutabazi et al. (2020) [87]	Over the years, the prevention of mother-to-child transmission of HIV/AIDS (PMTCT) guidelines have been adapted, but no strategies reported	None reported	None reported	None reported	None reported	None reported
Ryan et al. (2020) [109]	Comprehensive community mental health programme (CCMHP) A scale-up initiative for the general mental health policy implementation in Nigeria through public–private partnership in healthcare delivery	Absence of more clinical resources	Scale-up; reactive	None reported	Phone psychiatrists as needed; adding element	Community psychiatric nurses (CPNs) and community health extension worker (CHEWs)
Schneider and Nxumalo (2017) [97]	Re-engineering of primary healthcare (PHC)	To meet the needs and demands of each community health programme	Not reported; planned proactive	District managers, senior provincial managers, PHC facility managers, outreach team leaders, senior district official, subdistrict managers, PHC facility supervisors, professional nurses, environmental health officers	Health posts vs PHC re-engineering Roles of nongovernmental organizations were redefined Change in the method of payment of CHW New curricula and training processes; tailoring leadership and governance changes	Healthcare workers—specifically community-based workers
Stein et al. (2008) [106]	Incorporating counselling skills into the Practical Approach to Lung Health in South Africa (PALSA) PLUS model Ongoing onsite training provides emotional support	Given the limiting understanding of nurse counselling skills (i.e. they often threatened patients instead of making recommendation), nurses conceive counselling as “advice” that must be complied with rather than the patient feeling empowerment in decision-making	During the implementation of the PALSA PLUS programme and this evaluation; reactive	Not reported	Ongoing site training and counselling; adding elements—incorporation of a prayer into nurse-training sessions, as a means of accessing spiritual reserves for emotional support	Primary healthcare nurses
Wingfield et al. (2015) [113]	Innovative socioeconomic intervention against TB (ISIAT) strategy was evaluated under the community randomized evaluation of a socioeconomic intervention to prevent TB (CRESIPT) project Regular steering meetings, focus group discussions and contact in the health posts	Increase adherence and participation in the programme	Pre-implementation and implementation; proactive	Stakeholders + recipients	Contextual—increased the speed of bank transfers; substituting the funding structures	Target intervention group
Zakumumpa et al. [85]	ART scale-up Nonphysician cadre were prescribing antiretroviral therapy	The shortage of physician-level cadre was identified as a constraint	Scale-up; reactive	Individual practitioners	Implementation and scale-up activities; tweaking—nonphysician cadre were prescribing ART due to rapidly expanding patient volumes	Clinic/unit level, individual practitioner

### Implementation strategies

Eleven articles included in our review did not provide sufficient detail to adequately discern the strategies used to implement their health system guideline. 38 out of the 72 ERIC-defined implementation strategies were utilized across all 41 studies. A small number of reported implementation strategies were determined by consensus to fall under two separate ERIC categories and were coded as such. Studies reported a range of one to eight strategies to implement their health system initiative, with an average of four distinct implementation strategies. Conducting ongoing training was identified as the most frequent implementation strategy (*n* = 11), followed by building a coalition (*n* = 8), use of advisory boards and workgroups (*n* = 6), conducting educational meetings (*n* = 6) and developing educational materials (*n* = 5). The least prevalent ERIC-defined implementation strategies included, but were not limited to, revision of professional roles (*n* = 2), alterations of incentives/allowance structure (*n* = 2), assessments for readiness and identification of barriers and facilitators (*n* = 1), and tailoring of strategies (*n* = 1). A full breakdown of all 38 implementation strategies and their frequencies can be found in Table 4. None of our included studies explicitly reported the use of a theoretical/conceptual framework to guide their selection of implementation strategies.

**Table 4:** Implementation strategies coded using the ERIC framework

ERIC category	Occurrences	Implementation strategies (author/year)
Conduct ongoing training	11	Conduct ongoing training (Ejeta et al. 2020 [107]; Lovero et al. 2019) [93] Training sessions (Xia et al. 2015) [89] Education and retraining (Callaghan-Koru et al. 2020) [86] Training (Kavle et al. 2018 [114]; Rahman et al. 2020 [105]) Clinical training (Sami et al. 2018) [102] Staff in primary care settings to receive training and supervision for basic mental health screening, diagnosis and treatment (Lovero et al. 2019) [93] Trained in key modules of WHO’s Mental Health Gap Action Programme Intervention Guide (Ryan et al. 2020) [109] Capacity-building of medical doctors (MDs) through high-intensity training and onsite supervision (Miguel-Esponda et al. 2020) [69] Develop and conduct tailored training for nurse midwives and clinical officers at dispensaries (Mkoka et al. 2014) [94]
Build a coalition	8	Establishment of task teams, appointing leaders and NGO partnerships to lead and manage change (Schneider and Nxumalo 2017) [97] The programme proposal was presented and discussed with the staff. With the approval of the team, the process was gradually implemented (Bergerot et al. 2017) [79] Mutual promotion between national and local policies (Zhou et al. 2019) [67] Partnering with community associations (Lavôr et al. 2016) [111] Support for referrals to specialist services (Miguel-Esponda et al. 2020) [69] Collaboration and support from international development partners; national procurement planning and coordination (Rahman et al. 2020 [105]) Establish primary healthcare (PHC) network in one district of each province in the first year (Moshiri et al. 2016) [95] Integrated into curative health services provided by the national government (Gueye et al. 2016) [108]
Develop educational materials	7	Develop educational materials (Ejeta et al. 2020 [107]; Andrade et al. 2017 [75]) Standardization of materials (Roman et al. 2014) [66] New training methods to create a more harmonized and educated workforce (Kihembo et al. 2018) [57] Written policy statement that is routinely communicated (Kavle et al. 2018) [114] Designed training materials (self-reading, teaching aids and videos) based on the principles of participatory learning (investigators of WHO Low Birth Weight [LBW] Feeding Study Group, 2016) [110] Treatment guidelines (Rahman et al. 2020) [105]
Use of advisory boards	6	Stakeholder engagement (Roman et al. 2014) [66] Community groups and activist and healthcare professional acceptance and support; obtaining assistance from community health workers (Mutabazi et al. 2020) [87] Development of a chlorhexidine technical working group (Callaghan-Koru et al. 2020) [86] Promote collaboration between healthcare staff, support groups and local community; orientation of national policy- and decision-makers, management and community committees (Kavle et al. 2018) [114] Strategic planning workshops (Sami et al. 2018) [102] Elicited feedback on any site-specific concerns not addressed by the proposed system (Halpern et al. 2010) [77]
Conduct educational meetings	6	Education to healthcare providers (Roman et al. 2014) [66] Health education sessions (Kavle et al. 2018) [114] A national training and feedback session (Halpern et al. 2010) [77] Participatory community meetings for information (Wingfield et al. 2015) [113] Conducting educational activities for adherence to directly observed therapy (DOT ; Lavôr et al. 2016) [111] Countries conducted orientation meetings (Bryce et al. 2005) [58]
Distribute educational material	5	Distributed educational material (Ejeta et al. 2020) [107] Routinely distributed policy statement (Kavle et al. 2018) [114] Designed training materials (self-reading, teaching aids and videos) based on the principles of participatory learning (investigators of WHO LBW Feeding Study Group, 2016) [110] Printed educational materials for clinical decision-making (Miguel-Esponda et al. 2020) [69] Treatment guidelines (Rahman et al. 2020) [105]
Promote network-weaving	5	Leading and managing change—establishment of task teams, appointing leaders and NGO partnerships (Schneider and Nxumalo 2017) [97] Collaboration between national reproductive health programmes and national malaria control programmes (Roman et al. 2014) [66] Coordination of Community Cadres within the health system (Shelley et al. 2016) [99] Multi-department participation and collaboration to better implement the national essential drugs policy (Li et al. 2015) [112] Targeted interactions of PHC designers with local actors shaped a wide network of friends before the implementation phase (Moshiri et al. 2016) [95]
Conduct educational outreach visits	4	Education to healthcare providers (Roman et al. 2014) [66] Ongoing onsite training provides emotional support (Stein et al. 2008) [106] Monthly visits from a member of the working group to validate reports and address any implementation issues (Halpern et al. 2010) [77] Developed management and training capacity in a limited number of districts (Bryce et al. 2005) [58]
Access new funding	4	Ensuring financial stability (Roman et al. 2014) [66] Financial guarantee from the central government (Zhou et al. 2019) [67] Distribution of amoxicillin by UNICEF (Rahman et al. 2020) [105] Programme financing (Miguel-Esponda et al. 2020) [69]
Stage implementation scale-up	4	Implementation scale-up (Callaghan-Koru et al. 2020) [86] Pilot project was evaluated first; when it was deemed successful, the guideline was implemented at all existing care sites, one site at a time (Halpern et al. 2010) [77] End of one phase was marked with a review meeting with the objective of synthesizing early implementation experience and planning for expansion (Bryce et al. 2005) [58] Policies were implemented in a series of stages (Leethongdee, 2007) [83]
Develop and organize monitoring systems	4	Surveillance system and performance and monitoring framework (Kihembo et al. 2018) [57] Programme monitoring (Kavle et al. 2018 [114]; Bryce et al. 2005) [58] Following each assessment, quality improvement plans are generated and provided to facility managers to guide their improvement actions (Muthathi et al. 2020) [96]
Develop resource-sharing agreements	4	Management of resource availability; commodities/resources availability (Roman et al. 2014) [66] Distribution of medical commodities (Sami et al. 2018) [102] Ensuring medication supply (Miguel-Esponda et al. 2020) [69] Supply and distribution of amoxicillin dispersible tablets (Rahman et al. 2020) [105]
Provide clinical supervision	4	Provide clinical supervision (Sami et al. 2018 [102]; Lovero et al. 2019 [93]) Staff in primary care settings to receive training and supervision (Lovero et al. 2019) [93] Capacity-building of MDs through high-intensity training and onsite supervision (Miguel-Esponda et al. 2020) [69]
Develop a formal implementation blueprint	3	Five-year strategic plan with workplans (Kihembo et al. 2018) [57] Planning and early implementation, developed national strategy and plan (Bryce et al. 2005) [58] Network expansion plan; required budget was estimated and suggested to government; establish PHC network in one district of each province in the first year (Moshiri et al. 2016) [95]
Develop and implement tools for quality monitoring	3	Develop and implement tools for quality monitoring (Ejeta et al. 2020) [107] Standardization of materials; performance assessments (indicators); monitoring and evaluating (Roman et al. 2014) [66] Monitoring through a health information system (Miguel-Esponda et al. 2020) [69]
Change physical structure and equipment	3	Provide essential equipment and supplies; build/improve infrastructure for service delivery (Mkoka et al. 2014) [94] Availability of basic equipment (Rahman et al. 2020) [105] Providing containers to collect sputum and other inputs in the laboratory (Lavôr et al. 2016) [111]
Use train-the-trainer strategies	2	Train-the-trainer strategies (Ejeta et al. 2020 [107]; Kihembo et al. 2018) [57]
Recruit, designate and train for leadership	2	Recruit, designate and train for leadership (Ditlopo et al. 2011) [91] Top-down supervision from the central government (Zhou et al. 2019) [67]
Promote adaptability	2	Development and adaptation of guidelines to make them specific for low-income contexts (Callaghan-Koru et al. 2020) [86] Adapted the guidelines to their national context (Bryce et al. 2005) [58]
Alter incentive/allowance structures	2	Conditional cash transfers to reduce TB vulnerability; incentivize and enable care (Wingfield et al. 2015) [113] Alter incentive/allowance structures (Ditlopo et al. 2011) [91]
Centralize technical assistance	2	Centralize technical assistance (Andrade et al. 2017) [75] Development of new systems (integrating human resources, financing, etc.) that provided alignment across various departments (Schneider and Nxumalo 2017) [97]
Conduct local consensus discussions	2	Stakeholder engagement (Roman et al. 2014) [66] Targeted interactions of PHC designers with local actors shaped a wide network of friends before the implementation phase (Moshiri et al. 2016) [95]
Involve executive boards	2	Trained key decision-makers and built government commitment (Bryce et al. 2005) [58] Integrated care into health services provided by the national government (Gueye et al. 2016) [108]
Involve patients/consumers and family members	2	Initiated groups/forums such as Mother to Mother service—where trained mothers living with HIV provided psychosocial support to pregnant women and mother of babies diagnosed with HIV (Mutabazi et al. 2020) [87] Participatory community meetings (Wingfield et al. 2015) [113]
Obtain and use patients and family feedback	2	Obtain community acceptance (Shelley et al. 2016) [99] Community dialogue and action days (Kavle et al. 2018) [114]
Organize clinical implementation team meetings	2	Support groups; mentorship and support (Kavle et al. 2018) [114] Elicited feedback on any site-specific concerns not addressed and encouraged system buy-in among the individuals who would ultimately implement the system (Halpern et al. 2010) [77]
Revise professional roles	2	Reallocation of roles and responsibilities (Schneider and Nxumalo, 2017) [97] Stream linking tasks and roles to expand treatment and care for HIV (Mutabazi et al. 2020) [87]
Provide ongoing consultation	1	Supervision/support system (Shelley et al. 2016) [99]
Capture and share local knowledge	1	Capture and share local knowledge (Andrade et al. 2017) [75]
Use other payment schemes	1	A new public health insurance scheme which provides treatments within a defined “core” benefits package to registered members for a co-payment (Leethongdee 2007) [83]
Provide local technical assistance	1	Between visits, throughout the implementation process, working group members were available for technical consultation (Halpern et al. 2010) [77]
Make training dynamic	1	Training as a facilitated, interactive and more hands-on approach to learning; integrating learning and practice clinical work allow for feedback/revisions/clarifications (Stein et al. 2008) [106]
Make billing easier	1	Institution flow for timely funding (Lavôr et al. 2016) [111]
Inform local opinion leaders	1	Built government commitment to move forward (Bryce et al. 2005) [58]
Assess for readiness and identify barriers and facilitators	1	Baseline assessment (Kihembo et al. 2018) [57]
Change record systems	1	Change record systems (Ejeta et al. 2020) [107]
Create new clinical teams	1	Deploy health workers (Mkoka et al. 2014) [94]
Tailor strategies	1	Tailor strategies to local context (Andrade et al. 2017) [75]

### Outcomes of interest

Table 5 summarizes the outcomes of interest and key results of included studies. Nineteen articles reported the involvement of key stakeholders in various aspects of their design and implementation processes. Stakeholders varied from frontline healthcare workers to policy-makers, government organizations and nongovernmental organizations (NGOs). Outcomes of interest were related primarily to documenting and evaluating the implementation process, as well as the impact of the guideline on the health system (*n* = 39). These included assessing the barriers to and enablers of implementation, eliciting end-users’ experiences and perspectives, monitoring system and service changes, evaluating resource use, identifying future steps and comparing guideline expectations to real-world impacts. Additionally, one article explicitly specified the documentation of an implementation framework as an outcome of interest. Patient-level outcomes were noted as an indicator of success and included measuring health outcomes and quality of care delivery (*n* = 3). While many outcomes of interest were indicators of the overall success of the health system guideline integration, there were no outcomes of interest specifically reported as related to adaptations.

**Table 5:** Summary of results and outcomes

Author/year	Stakeholder involvement	Outcomes of interest	Outcome measures	Key results	Author conclusions/future directions
Amaral et al. [82]	None reported	Factors associated with the policy adoption	Data from state secretariats of health	New health interventions tend to be initially adopted by those who need them Smaller and more distant municipalities were less likely to have IMCI	It is necessary to define health policies in each state that promote the strategy in higher-risk municipalities
Andrade et al. (2017) [75]	Pan American Health Organization consulted on data collection methods Stakeholders involved in implementation included; Government of the State of Minas Gerais; Government of Santo Antonio do Monte; The National Council of Health Secretaries	Macro processes of attention to chronic conditions model (ACCM) Health outcomes associated with primary healthcare (PHC)	Household surveys and medical records Interviews Focus groups	Increase in community health agent visits Increase in individuals using public health services only among those with diabetes A decrease in doctor visits for individuals with diabetes	Having a unified health system as the main provider of primary care in small municipalities was important Establishing a PHC network in small municipalities was important Importance in implementation of the macro process Screening patients to receive treatment at different care levels
Armstrong et al. (2014) [90]	Reproductive and child health coordinators, a district laboratory technician, a district nursing officer, district medical officer (DMOs), health secretaries, and zonal maternal and perinatal death reviews (MPDR) medical officers were informants who were professionally involved in MPDR	The role and practices of MPDR in district and regional hospitals Key stakeholders’ involvement in and perspectives regarding the MPDR process	Interviews	Implementation of MPDR was dysfunctional The system still faces a number of challenges, most of which may be related to a lack of clarity in its intended purpose	It is unwise for providers to disengage Facility-level reviews are an important iterative learning process that should remain the core of any effort to improve care in health facilities Should Tanzania wish to change the MPDR system at the local level, evaluation, training and supervision are recommended
Bergerot et al. (2017) [79]	None reported	Patients’ distress, anxiety, depression and quality of life	Distress thermometer Hospital anxiety and depression scale Functional assessment of cancer therapy Structured questionnaire	The prevalence of distress was high compared with developed countries	Promote the development of strategies that favour equity in cancer care and that offer interventions in a timely manner Measures used were adequate for the identification of patients’ needs throughout the continuum of cancer The development of this screening programme achieved the goal of better meeting the psychosocial needs of cancer patients
Blanco-Mancilla (2011) [84]	None reported	Effectiveness of policy implementation	Interviews Newspaper articles Official documents Online news services and publications	Effective implementation in terms of access and capacity shows very different experiences between the policies analysed More than half of the total number of primary health centres managed by the department of health were still not certified to treat policy beneficiaries, seriously affecting access to services	These policy recommendations may help to improve implementation of the policies, as well as other new or current policies either in Mexico or in other countries
Bryce et al. (2005) [58]	None reported	Compare findings of the Multi-Country Evaluation of IMCI Effectiveness, Cost and Impact (MCE-IMCI) relative to the programme expectation reflected in the IMCI impact model	12 country assessments In-depth studies at five sites Cross- site analysis	The quality of trained IMCI workers was better than that of the untrained workers, even with no supervision Improving the quality of care in first-level government health facilities was not sufficient to increase low utilization levels The model reflected issues directly related to service delivery, but showed insufficiencies with other aspects of the health system such as transition pathways from policy and strategy to operations, human resource issues including supportive supervision, financing and ensuring an equitable coverage of interventions	New attention to child survival, new leadership in key organizations, and a focus on achieving the Millennium Development Goal of reducing child mortality by two thirds all provide the impetus to move quickly, forcefully and in new ways to achieve universal coverage with proven child survival interventions
Callaghan-Koru et al. (2020) [86]	Ministry of Health and Family Welfare (MOHFW)’s IMCI unit acted as the resource team coordinating scale-up A group composed of stakeholders from government, academia and NGOs to made policy recommendations and provided guidance A local pharmaceutical company supplied single-dose bottles Local NGOs were contracted to coordinate the training of providers in each district	Facilitators and barriers with respect to the institutionalization and expansion stages	Interviews Focus groups	Documenting facilitators and barriers with respect to scale-up of chlorhexidine (CHX) policy (see Barriers/Enablers Table) Strong leadership was a huge success factor Public system was not evaluated given the complexity and limited regulatory control in this sector Scale-up benchmarks would be useful approaches for identifying key institutionalization changes Changes should be adapted to reflect the full structure of the health system CHX counselling and distribution have not been routinely implemented in antenatal care expansion, suggesting that distinct plans and implementation strategies are needed to achieve goals within the two scale-up dimensions	The scale-up of CHX in Bangladesh was influenced by a range of factors from all five CFIR domains
Carneiro et al. (2018) [100]	None reported	Strategy performance	Population coverage estimated by primary care teams Proportion of live births to mothers with/without prenatal consultations Hospitalization rates due to primary care-sensitive condition Infant mortality rate	Resulted in changes to the management and control models used in the region, and introduced universities to the process The proportion of live births to mothers with/without prenatal consultations increased by 97% on average, predominantly with seven consultations or more and reducing the proportion of live births to mothers without prenatal visits The infant mortality rate achieved a downward trend	The results indicated the contribution of the more physicians in Brazil programme (MPBP) towards improving primary care based on the selected indicators
Costa et al. (2014) [101]	None reported	Indication of coverage Evidence of change and impact	Home visits made by doctors Requested exams of clinical pathology Referrals to specialists, and individual care provided by nurses Number of hospitalizations due to conditions that would respond to outpatient care (i.e. indicator of impact)	A majority of municipalities maintained the coverage level verified in 2004 One municipality presented strong indications of change in 2008 and was reclassified as moderate so as to allow the conduction of the statistical test An increase of 50% in the proportion of municipalities classified as high-impact More coverage compared with previous periods Lower rates of morbidity The proportion of municipalities with the expected number of requests remained small	There should be revision of work processes in Family Health Strategy (FHS) units, and a more in-depth investigation of the factors driving the small number of medical home visits, referrals to a specialist, requests for clinical pathology exams, and limited nursing care in relation to the number of medical consultations
Ditlopo et al. (2011) [91]	None reported	The implementation and perceived effectiveness of a rural allowance policy The motivation and retention of healthcare professionals (HCPs) in rural hospitals	Interviews Policy review	Partial effectiveness of rural allowance in recruitment Almost all policy-makers, hospital managers and HCPs consistently perceived the rural allowance to be divisive because it excluded junior nurses Remoteness of the area not considered Financial incentives alone were insufficient	Retention strategies that combined financial and nonfinancial incentives are likely to be more effective than increased remuneration alone, but these would need to be tailored to individual country contexts
Doherty et al. (2017) [92]	Stakeholders were involved in determining the reasons and sustainability of the policy	Impact of Prevention of mother-to-child transmission of HIV/AIDS (PMTCT) Option B+ implementation on the Uganda health system	Interviews Focus groups	Financial sustainability of the programme was a recurring theme because of funding insecurity Senior stakeholders voiced concerns about the health system’s readiness to adopt the policy and the rapid pace of scale-up	Uganda has achieved success in scaling up access to ART and reducing the number of children newly infected with HIV If ongoing investments and technical support for the HIV/AIDS response in Uganda are not allocated to strengthen the health system across programme areas, a significant opportunity may be lost
Ejeta et al. (2020) [107]	City/town health offices Sub-city and district offices Community leaders Regional health bureaus Ethiopian Federal Ministry of Health Members of the SEUHP programme Health centres	Lessons learned Challenges to implementation	Interviews Document review	The pilot test enabled the urban health extension professionals (UHE-Ps) to comprehensively focus on the 15 health service packages Use of tally sheet helped collect high-quality data and report it to city/town health offices Systematic categorization of households, based on their economic status and health service needs allowed for effective time management and delivery of services to vulnerable populations	Plans are made to scale up the programme to major cities
Febir et al. (2015) [103]	None reported	Perceptions of healthcare workers (HCWs) regarding the issues faced	Interviews	Implementation faced challenges given the weak health systems in most developing counties The perceptions of frontline HCWs on the accuracy and need for the guideline together with the capacity of health systems to support implementation played a crucial role Guidelines on financing of diagnostics and treatments are influencing clinical decision-making in this setting	Further research is needed to understand the impact of the National Health Interview Survey (NHIS) on the feasibility of integrating test-based management for malaria of the IMCI guidelines Findings suggest that the problem is heightened by beliefs and habits of frontline health staff in health facilities in developing countries that are used to presumptive treatment and perceive every fever to be malaria
Gueye et al. (2016) [108]	None reported	Ways in which countries have implemented elimination programmes The development and adoption of programmes How programmes operated within their context	Review of case study reports	Malaria programmes did not show a high level of capacity for anticipation of threats to elimination There were many examples of major development projections that combined a potential for increased receptivity and vulnerability Monitoring and evaluation included monitoring programme outputs and evaluation of impact	Global malaria eradication will require well-managed malaria programmes providing high-quality implementation of evidence-based strategies, founded upon strong surveillance and response strategies tailored to the subnational level transmission context Adequate funding and human resources to sustain malaria elimination and prevention of reintroduction is also required
Halpern et al. (2010) [77]	Stakeholders agreed on the ideal system for Guyana	Implementation strategy Benefits of monitoring national programmes	Cross-sectional reports Cohort analysis reports Monthly visits from a member of the working group Patient charts and registers	A large discrepancy was found between the data provided in the cross-sectional reports submitted prior to the use of the PMS and the data from those submitted after its implementation 79% of a combined national cohort who started ART were alive and on first-line ART regimens. After 6 years, 58% of the first cohort of ART patients in the country were alive and on ART, with only 8% patients on second-line regimens	The lessons learned during implementation can be used to better inform other countries in the region in need of information systems that can both improve patient care and produce high-quality data to inform programmatic and policy decisions
Investigators of WHO Low Birth Weight (LBW) Feeding Study Group (2016) [110]	None reported	Assessment of facilities, supplies and equipment Assessment of quality of care Assessment of knowledge, clinical skills and counselling skills of HCPs	Observation visit by expert paediatrician Written test Five objective structured clinical examinations Interviews with HCPs for feedback (in post-implementation phase only)	30% of nurses reported a significant increase in their workload following implementation of the guidelines No significant change in key practices like early initiation of breastfeeding, exclusive breastfeeding and prelacteal feeding Resulted in significant improvement in the knowledge and skills of HCPs and mothers and were instrumental in promoting positive health behaviour at hospital discharge	Needed additional efforts on part of HCWs/additional staff and efforts to promote generic early feeding practice
Kavle et al. (2018) [114]	Ministry of Health UNICEF Kenya Partnerships NGO	Implementation experience of Baby-Friendly Community Initiative (BFCI) Successes, challenge, and lessons learned Opportunities for integration Discuss the future and next steps	Review of key governmental programme documents Implementation monitoring	Coverage of BFCI was high and it surpassed the government target of 28% of all “community units implementing BFCI” by 2016/2017 Improved early initiation of breastfeeding and exclusive breastfeeding (EBF) were notable during and after implementation for a 3-month period	Buy-in from national leaders is key Mentorship by trainers played a key role Social mobilization efforts promote EBF Implementation can motivate early and frequent antenatal care (ANC) attendance, encourage attendance to health facility for childbirth and may improve immunization uptake
Kihembo et al. (2018) [57]	UKAid Department for International Development United Nations Central Emergency Response Fund (CERF) Newborn, adolescent and child health United States Agency for International Development (USAID) Centers for Disease Control and Prevention (CDC)	Document the IDSR implementation framework Evaluate planning and monitoring Understand the design and organization Understand the logistics and resources deployed in the process	Pre- and post-training scores Review of published and unpublished guidelines Review of preparedness and response protocols Review of training documents Interviews Meeting minutes	Through a coordinated partner support and response, funding, which was not primarily earmarked for IDSR implementation, was mobilized and harnessed to achieve nationwide equipping of multidisciplinary district teams with skill sets and tools necessary for performing relevant functions	A collaborative effort results in a coordinated significant impact on public health The revitalization of the IDSR programme highlights unique features which can be easily adopted and applied by other countries that wished to strengthen their IDSR programmes
Lavôr et al. (2016) [111]	None reported	Degree of implementation	Interviews with nurses Record book of symptomatic respiratory patients Record book and monitoring of TB cases Patient charts Treatment form Monthly report activity	In bacteriological diagnosis, classification was partially implemented Only bacilloscopies for follow-up treatment are carried out in 100% of basic health units (BHU) There was no relationship between the degree of implementation and effectiveness of the programme Political organization in the implementation of the direct observation of therapy (DOTS) strategy was impaired and weakened by its implementation	Mobilized community partners with HCPs can be organized in support of a cause and build their own strategies of actions to strengthen public health policies, through the inclusion in the formal social control agencies The DOTS strategy was classified as partially implemented in the BHU studied
Leethongdee (2007) [83]	None reported	Influences of implementation	Interviews Focus groups Documentary analysis	Main changes focused on the role of public organizations, and tensions between the old and new administrative structures The choice of funding mechanism was an important area of local discretion Many respondents, especially at lower levels, had a poor understanding of the purchaser/provider split about to be implemented in the Thai system, which highlighted the huge shift in culture that would be required in the new system There was a macro-level problem concerning the distribution of finance and the workforce across the nation There was a micro-level problem concerning the distribution of resources by contracting units for primary care (CUPs) to hospitals and health centres	There was a cycle of policy prescriptions, local adaptations and higher-level policy revisions that affected several aspects of the reforms and particularly the financing mechanism, which resulted in the lower-level actors having the most impact
Li et al. (2015) [112]	None reported	Impact of essential drug policy on primary care services Effectiveness of implementing essential drug policy	Field observation Main operation indicators	Implementation was very stable The health administrative departments should strengthen the choice, confirmation, assessment and control of distribution companies, establish the industry standards of drug distribution industry as soon as possible, and improve the access threshold	Through the investigation of grassroots medical institutions, we can determine the principles, varieties and prices of specifically supplied drugs, and the state can designate specialized manufacturers for drug manufacturing and government can provide financial subsidies
Lovero et al. (2019) [93]	None reported	The procedures for stepped care management Perceived challenges to implementation HCP training HCP experiences of managing mental illness	Interviews Questionnaires	Mental health screening should be conducted by nurses for all patients at PHC facilities Mental healthcare referrals should be made within clinic to MHPs and/or to other facilities based on case severity and availability of mental health personnel within clinic	There is a lack of training and consistency in the uptake of roles and responsibilities by nurses and MHPs Improved district-level administrative coordination, mental health awareness and financial resources are critical to the success of integration efforts
Miguel-Esponda et al. (2020) [69]	None reported	The extent to which the programme activities have been integrated into the organization and the PHC clinics	Sociodemographic and clinical characteristics Interviews	Challenges to delivery of services within the programme included time constraints coupled with the many competing priorities present at the clinics, and the limited availability of specialists to provide mentorship to MDs All MDs and clinical supervisors perceived a need for more involvement of either psychologists or psychiatrists to improve the training and supervision and also to advise on difficult cases	Integration of mental healthcare services in PHC will require improved financing and resource management of PHC and specialist services, ongoing capacity-building, the development of effective referral systems, further development of community-based services, and linking of PHC with locally relevant social interventions
Mkoka et al. (2014) [94]	Involved in implementation	Exploring the experience of respondents in implementing emergency obstetric care (EmOC) Perceived role of partners in EmOC implementation	Interviews Focus groups Facility survey Documentary reviews	Council health management team (CHMT) took the lead and worked with team spirit There was increased demand for services There was resource scarcity in terms of skilled HCPs, funds and time Working with competing needs Acknowledging importance of partners, partially because they play different roles A need for clear working arrangements A desire for community participation Progressing towards better service	Advocates working together in partnerships to govern implementation To have effective partnerships, the roles and responsibilities for each actor should be clearly stipulated in a clear working framework within the district health system
Moshiri et al. (2016) [95]	Because the implementation requirements, including staffing, structure and funding, were in the hands of the deputy for health, there was limited collaboration with the other sections of the MOH	Details of implementation	Interviews	The implementation approach better corresponded with a top-down approach that realizes policy change versus a hierarchical process	Existence of a working PHC network served as proper infrastructure for its implementation
Mutabazi et al. (2020) [87]	Stakeholders included the United States President’s Emergency Plan for AIDS Relief; Global Fund to Fight AIDS, Tuberculosis and Malaria; USAID; CDC; International NGOs	Experiences involved in daily activities	Interviews Self-administered questionnaire	Agreement on the importance of guideline integration Frontline HCPs experienced high workloads, high staff turnover and lack of infrastructure Additional assistance from HCP and nurses was essential for support Increased testing from the implementation of PMTCT programme showed a reduction in diagnosed HIV/AIDS in children	Addressing the challenges of integration of PMTCT will help in eliminating mother-to-child transmission of HIV/AIDS
Muthathi et al. (2020) [96]	Involved in design and implementation	Policy context, rationale and philosophy Intergovernmental relationships, perceptions of roles and responsibilities in implementation ICRM programme resourcing Implementation progress, challenges and constraints	Interviews	The central theme was the imperative to improve the quality of PHC in preparation for implementation Four themes emerged related to structural context: contestations about roles and responsibilities; weak intergovernmental relationships; enabling local leadership; and insufficient resourcing of the ICRM programme Three themes emerged related to specific context: gaps in the existing NCS; insufficient policy coherence; disjuncture between the NCS and ICRM programme	The design of any health reform should consider policies or initiatives that ensure coherence and the availability of resources Major change initiative requires involvement of all relevant policy actors in design and implementation Clear communication strategies and ongoing monitoring and evaluation are prerequisites for the success of policy implementation
Pyone et al. (2017) [104]	Qualitative research was carried out using semi-structured interviews with 39 key stakeholders from six countries in Kenya	The implications of the implementation of the free maternity services (FMS) policy on health system governance Strength of the implementation programme	Semi-structured interviews Institutional analysis as a theoretical framework	The newly introduced formal institutional (re)arrangements were unclear Implementers faced challenges of accountability, especially adherence to the FMS policy When resources were constrained, HCPs were less likely to be accountable, as they were not provided with the resources to work	There were discrepancies between formal and informal rules which created a misalignment of incentives for policy implementation Aligning the objectives of the implementers with new policies, corresponding institutional (re)arrangements, enforcement mechanisms and incentives is crucial
Rahman et al. (2020) [105]	Stakeholders discussed the challenges and opportunities for implementation of the WHO recommendations that emerged from the study	Facilitators and barriers to implementation	Interviews Documents analysis	Advocacy initiatives should be undertaken to promote policy revisions Training and instructions should be provided Incomplete policy adoption can be attributed to insufficient coordination among divisions; lack of central procurement of amoxicillin dispersible tablets (DT); and perceptions of the efficacy of antibiotics and formulations at the national and district levels	Significant progress occurred, but key challenges remain at the national and subnational levels, contributing to slow adoption of the WHO recommendations for the case management of childhood pneumonia and possible serious bacterial infection (PSBI) using amoxicillin DT
Roman et al. (2014) [66]	Stakeholders helped inform the development of key informant interview guides Qualitative data were collected through in-depth interviews among key stakeholders at the national level	Promising practices/strategies that have support programming success Implementation barriers Lessons learned	Secondary data (literature review) Interviews	*Integration*—strengthening and creating national groups (stakeholders) *Policy*—in line with WHO guidelines and also interpreted in a similar manner across health systems *Commodities*—availability in drug resources and stock *Quality assurance*—assessment tools to monitor progress and alleviate barriers at the time *Capacity-building*—successful when focused on pre-training and in-service training *Community involvement/engagement*—linking community- with facility-level care and promoting community engagement and knowledge about MIP programme *Monitoring and evaluating*—three case studies did implement this and caused challenges for national synthesis and reporting *Financing*—more dedicated support for MIP programme by advocating building of in-country awareness from community to national level	The timing affords countries the opportunity to reprioritize MIP programming to ensure effective technical oversight and programme management
Ryan et al. (2020) [109]	Meetings with the CBM mental health advisor for Nigeria; welfare officers from community-based rehabilitation centres, the Bishop of the Methodist Church Diocese of Otukpo in Benue State, the Benue state health management information systems officer, the Benue state director of public health and other state and local government officials	Environment and health system in which the programme functions History of the programme Programme model and conceptual framework Engagement with broader systems Programme resources and management Client characteristics Pathways to care Clinical interventions Medications Psychosocial interventions Accessibility of services Information systems used	Field visits Service utilization data	It is possible to leverage a public–private partnership (PPP) with not-for-profit partners to rapidly expand mental health services in primary care Coordinated efforts across primary, secondary and tertiary care is needed	More research is needed to document and evaluate PPPs for mental health in LMICS, with a focus on sustainability
Saddi et al. (2018) [88]	None reported	Perceptions about primary care access and quality (PMAQ) Organizational barriers to the implementation of FHS	Semi-structured interviews Questionnaires	Low organizational capacity influenced the perceived impact of the doctors, nurses and community HCPs Adherence to PMAQ at the front line follows a top-down pattern; 46% of HCPs reported that adherence was the result of the PMAQ being imposed by the municipal health secretary (SMS), and 26% of HCPs reported adherence was due to trying to improve service quality	More contextualized public policy or health policy research, focusing on frontline workers, could be implemented
Sami et al. (2018) [102]	None reported	Explain the main health system bottlenecks for implementation Barriers and facilitators Recommended solutions	Focus groups Direct observations Collection of variety of documents	See barriers and enablers in Table 7	Further research to improve the implementation of community- and facility-level newborn interventions in settings with ongoing conflict Understanding the feasibility of guidelines recommended in context would allow for specific adaptations and innovations
Schneider and Nxumalo (2017) [97]	None reported	Policy formation/adoption Reallocation of roles and responsibilities Development of new systems How change is led and managed	Interviews Observations Document review Routine and audit data	Negotiating a fit between national mandates, provincial and district histories and strategies of community-based services Defining new organizational and accountability relationships between CHWs, local health services, communities and NGOs Revising and developing new aligned and integrated planning, human resources, financing and information systems Leading change by building new collective visions, mobilizing political support and designing implementation strategies	Contributed to an understanding of leadership and governance functions in strengthening CHW programmes Suggest the need for multilevel frameworks that provide both direction and flexibility, allowing for emergence and negotiation Highlighted the multifaceted, negotiated and distributed nature of these functions, spanning analytical, managerial, technical and political roles Future work includes evaluating the implications of assessing or strengthening the leadership and governance of national CHW programmes
Sheikh et al. (2010) [98]	None reported	Perspectives of different groups of actors on their own participation in the implementation process	Interviews	Informed consent was seen as unwelcome obstacles Physicians typically followed unwritten rules that were based on their own clinical judgement and the best interest of the patient, not necessarily the guideline Lack of private rooms resulted in physicians disclosing confidential results in front of other patients	Contributed an understanding of health policy implementation in India from the “emic” perspectives of the various participant actors
Shelley et al. (2016) [99]	This process evaluation utilized interviews with a variety of stakeholders to explore perspectives and lessons from the first 6 months of community health assistant (CHA) deployment	Lessons learned Barriers to and facilitators of fidelity	Interviews	Community acceptance is essential to successful programme implementation Effective and reliable supervision is considered a cornerstone to success	Findings allowed the government to make informed decisions and adjustments prior to second deployment of CHAs
Stein et al. (2008) [106]	None reported	Perceptions of those involved in the programme Value of the training approach	Participants’ observations Interviews Focus groups	Training was interactive and effective Integrative training approach allowed for supervisory feedback A horizontal training approach facilitated the implementation process Training was effective and more likely to be effective within a health system framework which consistently provides PHC services Improved quality of care was seen in a range of illnesses Nurses were overstretched and many PHC clinics were understaffed	All levels of healthcare system teams should be engaged in programme implementation
Wingfield et al. (2015) [113]	Formative activities included consultations, focus group discussions and questionnaires conducted with the project team, project participants, civil society and key NGO stakeholders	Cash delivery strategy Cash transfer size Cash transfer timing Cash transfer conditions, levels and responsiveness	Performed an acceptability assessment Quantitative and qualitative data from participants, a civil society group of ex-patient community representatives, CRESIPT [community randomized evaluation of a socioeconomic intervention to prevent TB] project staff and local and regional Peruvian TB programme staff and coordinators	A novel TB-specific socioeconomic intervention proved to be feasible in an impoverished, urban environment and is now ready for impact assessment, including by the CRESIPT project Of potential cash transfers, 74% were achieved, 19% were not achieved, and 7% were yet to be achieved Of those achieved, 92% were achieved optimally and 8% suboptimally Cash transfer strategy should be tailored to household needs	Lessons from CRESIPT will aim to assist TB control programmes to effectively implement the recent global policy change of including socioeconomic support as part of TB control activities
Xia et al. (2015) [89]	Stakeholders were interviewed and surveyed	Service user views on integrated prenatal HIV, syphilis and hepatitis B testing (PHSHT) services Service users’ knowledge and satisfaction of PHSHT services Factors affecting how the integration of services was coordinated	Survey Routine monitoring Interviews Focus groups	Pregnant women had little knowledge of PHSHT services and found the service process to be long and complicated HIV tests were above the national standard, unlike syphilis and Hep B Lack of referral network between lab results resulted in significant delays	Conducting regular meetings between health agencies could improve information exchange Establishing a proper client referral system with an integrated information systems could help reduce redundancy Decentralization of services could help simplify process Facilitate task-shifting and community participation
Zakumumpa et al. [85]	None reported	Sustainability of ART scale-up implementation Access to ART medicines Interconnections in health system subcomponents	National survey of health facilities Organizational case studies	Access to ART medicines at the level of frontline health facilities were influenced by information systems, human resources, governance and leadership Failure to maintain basic ART programme records, owing to health workforce shortages, contributed to chronic ART medicines stock-outs	Health system strengthening interventions, especially targeting lower-level and rural-based health facilities, are recommended to promote ART programme sustainability
Zhou et al. (2019) [67]	Consultations with stakeholders	Formulation process, content and implementation issues	Interviews Open-ended surveys	Strategies to achieve the four policy objectives were unevenly covered Two action areas, namely “quality improvement” and “procedure and distribution of essential medicines”, were not covered The limited human resources made working part-time very common Considering policy operationality, targets, time frames and evaluation indicated were consistent with national ones, but mainly set for priority strategies	Solid evidence, high-level approval, involvement of multiple stakeholders, detailed and comprehensive arrangements in operational issues, and clear policy focuses will promote successful implementation of mental health policy

### Outcome measures

Outcome measures included interviews/focus groups (*n* = 29), document/policy analysis (*n* = 10), surveys/questionnaires (*n* = 9), health administrative data and medical records (*n* = 8), field visits/observations (*n* = 4), secondary data from literature/guideline reviews (*n* = 2), individual case studies (*n* = 2), clinical assessment tools (*n* = 1), performance assessment tools (*n* = 1) and patient observations (*n* = 1). A full breakdown of outcomes is presented in Table 5.

### Barriers and enablers related to implementation

Reported barriers to and enablers of implementation of health system guidelines were coded using the COM-B framework [54]. Barriers and enablers that were most frequently reported by identified studies were associated with physical (*n* = 36) or social (*n* = 22) opportunity. Physical opportunities are defined as the environmental context and resources, whereas social opportunities refer to the social influences, such as norms and cultural factors [54]. Financial constraints, access to resources, and training (or lack thereof) were persistent physical opportunity factors described. Language and communication, political instability and power imbalances are all examples of reported barriers or enablers related to social opportunities.

Implementation barriers and enablers related to psychological (*n* = 15) and physical capabilities (*n* = 19) were the second most frequently coded category in the COM-B framework. Physical capabilities describe the skills and abilities required, while psychological capabilities refer to the concepts of knowledge, memory, decision-making and behavioural regulation [54]. Identified articles reported barriers and enablers related to the knowledge about implemented guidelines, the emotional toll on frontline workers and the resistance to change. Physical capabilities included adapting training materials specific to the needs of end-users and ongoing training/mentorship with supervision.

Reflective (*n* = 4) and automatic (*n* = 10) motivations were the least often coded barrier and enabler in our review. Reflective motivation refers to the roles, identities and beliefs about consequences [54]. Resistance to or acceptance of change, trust in the guidelines, and defining role and responsibility attributes are all examples noted among the reflective motivation category. Automatic motivation refers to the emotion and reinforcement influencing target behaviour [54]. Dedicated commitment, enthusiasm and motivation to implement health system guidelines were reported as a barrier and/or enabler. A summary of the COM-B analysis can be found in Tables 6 and 7. A full breakdown of extracted and analysed data can be found in Additional file 4.

**Table 6:** Implementation barriers and enablers coded using the COM-B framework (summary table)

COM-B category	COM-B subcategory	COM-B definition	Frequency of occurrence	Examples of barriers and enablers
Opportunity	Physical	Environmental context and resources	36	○ Financial constraints and budgets ○ Physical resources to support guideline implementation (water lines, lack of transportation, etc.) ○ Need for extensive human resources ○ Stakeholder support and buy-in ○ Site check-ins ○ Training for end-users and stakeholders ○ Supportive policies and laws ○ Ensuring basic needs are met for workers to support motivation and reduce attrition ○ Local leadership ○ Incentives ○ Strategic implementation and operation plans
	Social	Social influences, norms, cultural, social pressures, conformity	22	○ Cultural context ○ Political instability/stability ○ Political commitment ○ Stigma (e.g. HIV+ mothers counselling other HIV+ mothers) ○ Power imbalances
Capabilities	Psychological	Knowledge, memory, decision-making, behavioural regulation	15	○ Knowledge of the guideline and its practices ○ Emotional toll on frontline clinicians working with vulnerable populations ○ Resistance to change
	Physical	Skills and abilities	19	○ Adapting training materials for all (e.g. adapting materials for those who are illiterate) ○ Hosting training meetings ○ Continued implementation through training, mentorship, supportive supervision and follow-up documentation
Motivation	Reflective	Roles and identity, beliefs about consequences and optimism	4	○ Resistance to/acceptance of change ○ Trust in guidelines ○ More clear definition of roles and responsibilities
	Automatic	Emotions and reinforcement	10	○ Enthusiasm and commitment to implementation ○ Motivation to implement and perform duties

**Table 7:** Barriers and enablers related to adaption (COM-B analysis—opportunities, motivation)

Author (year)	Opportunities	
	Physical	Social
Andrade et al. (2017) [75]	○ Unable to implement an electronic system (enabler)	○ None reported
Bryce et al. (2005) [58]	○ Adapting guidelines to context (enabler)	○ None reported
Gueye et al. (2016) [108]	○ Programme showed flexibility over time, as it was able to mobilize a large number of staff	○ None reported
Halpern et al. (2010) [77]	○ A technical working group is crucial to help develop the country-specific systems, oversee implementation, and adjust or deal with unexpected changes (enabler)	○ None reported
Leethongdee (2007) [83]	○ Created a new catchment area which increased the budget (enabler)	○ None reported
Rahman et al. (2020) [105]	○ Readiness of the health system to execute the policy (enabler/barrier)	○ Proactive leadership from national programmes, advocacy, technical and resource support from international development partners (enabler)
Stein et al. (2008) [106]	○ None reported	○ The spiritual adaptation/incorporation provided culturally appropriate support (enabler)
Wingfield et al. (2015) [113]	○ None reported	○ Strong multisectoral collaboration (enabler)

	Motivation	
	Automatic	Reflective
Stein et al. (2008) [106]	○ None reported	○ Nurses valued counselling skills that were built as an adaptation to the guideline (enabler)
Wingfield et al. (2015) [113]	○ None reported	○ Lack of available evidence, and thus deciding on the transfer amounts and timing was difficult (barrier)

### Barriers and enablers related to adaptation

Eight articles reported barriers and enablers related to adaptation of the health system guidelines. Of these, physical opportunities were the most commonly reported barriers and enablers, with articles describing the use of technical working groups to adjust and manage unexpected changes, ensure flexibility in initiatives, and create new structures/systems to facilitate local adaptation (*n* = 6). Strong multisectoral collaboration, proactive leadership and culturally appropriate support are all examples of barriers and enablers related to social opportunities that were reported (*n* = 3). Reflective motivation (*n* = 2) was the only other COM-B category captured in reported barriers and enablers with respect to adaptation by this review. This related to a lack of available evidence influencing choices and end-users valuing additional (and adapted) components to the initiatives.

### Quality appraisal

Twenty-seven articles ranked high (67–100%) in their quality assessments. Seven articles ranked medium (33–66%) and seven ranked low (0–32%) (see Table 1). Those with medium- and lower-quality scores often lacked details related to their study methods, resulting in an unclear understanding of the implementation and initiative fidelity. Results from these studies should be considered with this in mind.

## Discussion

This scoping review located, mapped and codified published literature exploring the adaptation and implementation of health system guidelines in LMICs to assess trends and identify potential gaps. Through the synthesis of available evidence, we were able to identify common strategies for adapting and implementing health system guidelines, related barriers and enablers, and indicators of success.

Overall, the most common type of implementation strategies used to facilitate the integration of health system guidelines involved education, training, clinical supervision and the formulation of working groups and advisory boards. Examples of education and training include the development of standardized educational materials, as well as national training and feedback sessions (see Table 4 for a breakdown of all examples). While this review can comment on the types of implementation strategies utilized, specific details such as the duration and dose of these techniques were largely underreported by the authors of the included studies (e.g. 1-day vs month-long workshops). The reported educational and collaborative implementation strategies are in direct alignment with current literature and support similar emerging themes in other healthcare and income settings [55, 56]. A recent review of techniques used to implement nursing practice guidelines across different health settings reveals that education-based strategies were almost always incorporated in the implementation plan [55]. Our findings are consistent with other works in LMICs cited in Imamarua et al.’s (2017) literature synthesis of implementation strategies to deliver maternal practice guidelines [56]. While these reviews identified the involvement of local opinion leaders in their implementation tactics, the formal creation of advisory groups (such as developing technical working groups) appears to be more common in health system-based implementation initiatives than in clinical practice guidelines. This could be reflective of the complex nature of health systems, social norms and values in local communities regarding decision-making, and the various actors that need to be thoughtfully and proactively engaged to facilitate implementation. Furthermore, included studies used an average of four implementation strategies, and less than half of potential strategies available to them (38/73 techniques defined by ERIC). Thus, our review highlights the potential need to leverage and combine a wider variety of implementation techniques to address known barriers to changes and to achieve policy/programme goals.

Though most of the included articles detailed rationales for implementing their targeted health system guidelines, the selection of implementation strategies did not appear to be guided by foundational knowledge, theory or conceptual frameworks. Further, only three studies applied a formal implementation plan [57–59]. Implementation science literature highlights the critical importance of identifying and tailoring implementation techniques to successfully transition evidence into real-world practice [60, 61]. Conducting behavioural analyses to identify barriers and facilitators can then be used to guide the selection of evidence-based strategies and to mitigate potential challenges while simultaneously amplifying promising facilitators [54, 60, 61]. Differing levels of available human and physical resources, political structures, professional roles and responsibilities, and cultural and religious practices are all salient and intersectional factors that need to be considered within an implementation plan for health system initiatives [62, 63]. These contextual factors are of particular importance to consider in potentially resource-limited settings to optimize strengths and attend to weaknesses [63]. One component necessary for building a resilient health system is an awareness of the current strengths and weaknesses within existing structures to inform practice and policy planning [64]. There are various evidence-based frameworks and taxonomies that provide structured and systematic processes to identify existing barriers and enablers in specific contexts [26, 28, 54, 65]. Existing tools such as the COM-B model can be used to help identify and map known implementation barriers and enablers and assist in selecting targeted techniques to influence change at the health system level [54]. The use of evidenced-based conceptual and theoretical frameworks could help to improve the selection of individualized implementation techniques and ultimately improve the successful integration of health system guidelines in LMICs.

Lack of consistent funding was a noted barrier to the implementation of health system guidelines. Despite this, only a handful of articles reported accessing new funding sources as part of their implementation plan [66–69]. While seven studies were conducted in an LIC setting, reports of financial constraints were not limited to those within LICs. Our findings suggest that securing implementation research funding is arduous, irrespective of a country’s income level. Recent work from Ritchie et al. [62] explored the challenges experienced among LMICs when translating maternal health evidence into practice and revealed that lack of health system funding was one of the most common barriers to evidence implementation in LMICs. This barrier, however, may not be unique to LMICs, with sustained funding being challenging even among high-income contexts [70]. As highlighted in the implementation science literature, this is of particular importance when considering the ability to sustain the delivery of health system guidelines beyond their initial implementation [71]. Partnering with NGOs was one strategy utilized by some of our included articles to help fund initiatives. However, while initial financial support may provide the necessary seed money and resources to help launch initiatives, projects without sustained sources of funding risk being shut down [70]. It is also noteworthy that over half of the included studies reported funding sources stemming solely from high-income funding initiatives (e.g. Irish Aid, Australian government funding, Canada’s International Development Research Centre), with only 11 studies utilizing funds from their local country (i.e. Brazilian Ministry of Education, China’s Medical Board). Financial commitments and sustained funding from health ministries is essential to supporting implementation efforts and facilitating the longevity and sustainability of moving evidence into practice and strengthening implementation of health system guidelines into the real-world context.

When stratifying our findings by WHO’s health system building blocks, it became clear that change at the health system level is often dependent on addressing all intersecting concepts. For example, a majority of our identified health system guidelines targeted the service delivery building block, and yet their related barriers included lack of financing, resources and/or leadership and government commitment. Guidelines that targeted the health workforce building block reported barriers specific to the lack of knowledge about the guidelines, human resources and funding. These findings highlight the intersectional nature of all health system building blocks and the critical need to look across components to facilitate successful system-level change. When exploring Rwanda’s great success in improving health outcomes, Sayinzoga and Bijlmakers [72] discovered that one of the key factors influencing their successes was the recognition of the need for multiple and interconnected health system initiatives to achieve set goals. Without accounting for this intersectional nature, initiatives are unlikely to be successful, resulting in wasted time and efforts [63]. Strengthening health systems requires purposeful planning and action across building blocks to enact reform across all health, social and political structures [73, 74]. Researchers and decision-makers are encouraged to incorporate WHO’s health system building blocks as a framework to identify essential elements that may require additional support during the implementation and adaptation of health system guidelines.

Our review revealed a dearth of reported information related to the adaptation of health system guidelines in comparison to implementation strategies. We could find only one study that reported having tailored its guidelines to the needs of the local context as an implementation strategy [75], and only 14 studies reported adaptation techniques. Adapting both health system and clinical practice guidelines is critical to enhancing applicability to the specific setting and to account for differing cultural, organizational and environmental factors [76]. Adaptation of these initiatives can lead to increased local uptake by engaging stakeholders and end-users throughout the process [76]. However, this customization must be carried out carefully to ensure the correct application of evidence and recommendations. Utilizing evidence-based adaptation frameworks provides systematic guidance to ensure that the required modifications are made while still honouring the authenticity of the guideline [76]. Unfortunately, the use of adaptation frameworks was not reported in any of our included articles. While some articles reported on their adaptation techniques, such as Halpern et al.’s [77] detailed description of the creation of a technical working group to adapt each guideline component, most articles did not provide sufficient detail of their processes or reasoning. Rigorous research is needed to explore and identify the most effective adaptation strategies to enhance a guideline’s applicability and uptake at the health system level and support the use of these strategies in practice.

The most frequent indicators of success when implementing and adapting health system guidelines were related to assessing contextualized barriers and facilitators, end-user experiences, and monitoring system changes. Choosing outcomes and indicators is still a debated topic within implementation science literature [8]. As this review identified, many distinguish implementation success by evaluating the process itself (i.e. challenges and successes). Limited studies reported on cost as a critical implementation outcome. Without evaluating implementation cost, sustainability of the health system change is difficult to discern. Conducting cost–benefit analyses and verifying areas of potential cost savings could provide decision-makers with further evidence to support the granting of sustainable funding for implementation of health system guidelines—a major barrier identified in this review.

An alternative ideology asserts success as being related to a health system guideline’s ability to achieve its recommended target and improve care [78]. Only a small proportion of studies included in our review compared guideline targets with real-world changes or leveraged patient-level outcomes to identify improvements in quality of life and health outcomes [75, 79]. The integration of patient- and population-level outcomes may be an important component in the evaluation of health system guidelines in LMICs, as an ultimate goal of a resilient and sustainable health system is to better serve patients and families. There are also a variety of evaluation frameworks that can help guide researchers in the selection of outcomes and indicators of success at the health system level [49]. While flexibility is necessary in evaluation plans, utilizing these frameworks can provide structure and evidence-based processes to ensure comparable outcomes are being selected and reported. This would allow for the streamlined comparison and shared learning across LMICs and could facilitate a more transparent understanding of key factors that drive successful implementation of health system guidelines.

The findings from our quality appraisal and the lack of detail that we were able to extract related to certain concepts (i.e. adaptation strategies) highlight the need to improve adherence to reporting guidelines within this body of literature. By following reporting guidelines in the dissemination of study findings, we can help increase the transparency and completeness of research initiatives [80], ensuring that articles contain the important components and active ingredients for their implementation and adaptation strategies, evaluation methods and health system initiatives. Without this information, it is difficult for readers to discern how implementation and adaptation plans were developed, the techniques employed, and the trustworthiness of findings [81].

## Limitations

It is important to consider our findings considering potential limitations. First, our search strategy was limited to reports published in English. We consulted with our knowledge users, who advised that they did not believe this would influence our review findings; however, we acknowledge that not all initiatives conducted in LMICs are reported in this language. This may also partly explain our finding that most initiatives were funded by HICs. Second, given the variation in how authors describe health system guidelines (e.g. recommendations, policies), we may not have captured all potentially relevant studies. Further, it is worth noting that authors may not uniformly use the term “adaptation” when referring to the concept definition adopted in this work. Variations in terminology could have impacted our identification and/or extraction of data. However, our search strategy was carefully developed by an experienced library scientist to mitigate such challenges and comprehensively capture pertinent studies.

## Conclusions

Identifying evidenced-based strategies to successfully move evidence into practice continues to be a growing and critical area of research. Health system guidelines are pivotal tools to optimize, strengthen and develop resilient healthcare infrastructures and provisions. This scoping review provides a comprehensive overview of published literature examining the adaptation and implementation of health system guidelines in LMICs. Our findings revealed the most common strategies for implementing health system guidelines in LMICs, including education, training, clinical supervision and formation of advisory groups. There is a need to explore the impact of leveraging and combining a wider variety of implementation techniques to achieve policy/programme goals. The reporting of adaptation strategies was an evident gap in this body of literature, highlighting the need for more primary research aimed at identifying effective adaptation techniques to enhance a guideline’s applicability and uptake at the health system level. Given the lack of theoretical frameworks identified in included studies, research teams can turn to established implementation and adaptation frameworks as a starting point to help guide their work. Furthermore, while the absence of sustained funding and financial commitments was identified as a salient barrier to the implementation of health system guidelines, there was a lack of studies reporting cost as an evaluation outcome. Future researchers are encouraged to consider conducting cost analyses to create a case for decision-makers to support the granting of sustainable funding for health system guidelines. Our findings suggest that more effort may be required across research, policy and practice sectors to support the adaptation and implementation of health system guidelines to local contexts and health system arrangements in LMICs.

## Supplementary Information

**Additional file 1.** Search strategy.

**Additional file 2.** Data extraction form.

**Additional file 3.** Acronyms.

**Additional file 4.** Barriers to and enablers of implementation categorized by the COM-B framework (opportunities category).

## Data Availability

All data generated or analysed during this study are included in this published article and its additional files.

## Abbreviations

LMICs: Low- and middle-income countries
LIC: Low-income country
HIC: High-income country
EPOC: Effective Practice and Organisation of Care
COM-B: Capability, Opportunity and Motivation Behaviour model
RAISE: Research to Enhance the Adaptation and Implementation of Health Systems Guidelines
