# Supplementary material for: Strategies to adapt and implement health system guidelines and recommendations: a scoping review
Source: Health Res Policy Syst. 2022 Jun 15;20:64. doi: 10.1186/s12961-022-00865-8 (PMC9202131; doi:10.1186/s12961-022-00865-8)
Supplement: Supplementary file 1 — Additional file 1. Search strategy. [file 12961_2022_865_MOESM1_ESM.docx]

**Additional file 1: Search Strategy**

| Medline (Ovid)  Date: August 18, 2020 | | |
| --- | --- | --- |
| Number | Query | Results |
| 1 | ((healthcare system? or health care system? or health system? or system wide or systemwide or system level or health service? or health system service? or health administration or service? delivery or delivery of health care or healthcare organi?ation* or health care organi?ation* or health organi?ation* or public health or community health or national health or federal health or local health or state health or provincial health or municipal health or county health or city health) adj3 (policy or policies or directive* or recommendation? or guideline? or guidance or program* or change? or innovat* or rule? or governance or initiative) adj10 (adopt* or adapt* or adhere* or "use" or follow* or implement* or change or integrat* or embed* or uptake or comply or complian*)).ab,ti. | 7732 |
| 2 | exp "health care facilities, manpower, and services"/ or "health care economics and organizations"/ or health services administration/ or "organization and administration"/ | 2917924 |
| 3 | Policy/ or Organizational Policy/ or Guideline/ | 32720 |
| 4 | (adopt* or adapt* or adhere* or "use" or follow* or implement* or change or integrat* or embed* or uptake or comply or complian*).ab,ti. | 8142900 |
| 5 | 2 and 3 and 4 | 4867 |
| 6 | 1 or 5 | 12547 |
| 7 | (best practice* or promising practice* or strateg* or support* or approach* or encourag* or foster* or advanc* or enforc* or incentiv* or enable* or facilitat* or advance* or context* or success or promot* or challenge* or barrier* or impediment or inhibit*).ab,ti. | 8251184 |
| 8 | 6 and 7 | 7791 |
| 9 | (Afghanistan or Bangladesh or Benin or "Burkina Faso" or Burundi or Cambodia or "Central African Republic" or Chad or Comoros or Congo or "Cote d'Ivoire" or Eritrea or Ethiopia or Gambia or Ghana or Guinea or Haiti or India or Kenya or Korea or Kyrgyz or Kyrgyzstan or Lao or Laos or Liberia or Madagascar or Malawi or Mali or Mauritania or Melanesia or Mongolia or Mozambique or Burma or Myanmar or Nepal or Niger or Nigeria or Pakistan or Rwanda or "Salomon Islands" or "Sao Tome" or Senegal or "Sierra Leone" or Somalia or Sudan or Tajikistan or Tanzania or Timor or Togo or Uganda or Uzbekistan or Vietnam or "Viet Nam" or Yemen or Zambia or Zimbabwe).ab,ti. | 486027 |
| 10 | (Albania or Algeria or Angola or Armenia or Azerbaijan or Belarus or Bhutan or Bolivia or Bosnia or Herzegovina or "Cape Verde" or Cameroon or China or Colombia or Congo or Cuba or Djibouti or "Dominican Republic" or Ecuador or Egypt or "El Salvador" or Fiji or Gaza or Georgia or Guam or Guatemala or Guyana or Honduras or "Indian Ocean Islands" or Indonesia or Iran or Iraq or Jamaica or Jordan or Kiribati or Lesotho or Macedonia or Maldives or "Marshall Islands" or Micronesia or "Middle East" or Moldova or Morocco or Namibia or Nicaragua or Palestin* or Paraguay or Peru or Philippines or Samoa or "Sri Lanka" or Suriname or Swaziland or Syria or "Syrian Arab Republic" or Thailand or Tonga or Tunisia or Turkmenistan or Ukraine or Vanuatu or "West Bank").ab,ti. | 411071 |
| 11 | ("American Samoa" or Argentina or Belize or Botswana or Brazil or Bulgaria or Chile or Comoros or "Costa Rica" or Croatia or Dominica or Guinea or Gabon or Grenada or Grenadines or Hungary or Kazakhstan or Latvia or Lebanon or Libia or libyan or Libya or Lithuania or Malaysia or Mauritius or Mayotte or Mexico or Micronesia or Montenegro or Nevis or "Northern Mariana Islands" or Oman or Palau or Panama or Poland or Romania or Russia or "Russian Federation" or Samoa or "Saint Lucia" or "St Lucia" or "Saint Kitts" or "St Kitts" or "Saint Vincent" or "St Vincent" or Serbia or Seychelles or Slovakia or "Slovak Republic" or "South Africa" or Turkey or Uruguay or Venezuela or Yugoslavia).ab,ti. | 444265 |
| 12 | (Africa or Asia or "South America" or "Latin America" or "Central America").ab,ti. | 183423 |
| 13 | (transitional adj countr*).ab,ti. | 160 |
| 14 | (lmic or lmics or (third adj world) or (lami adj countr*)).ab,ti. | 7841 |
| 15 | ((non government or non governmental) adj (organization* or organization*)).ab,ti. | 2216 |
| 16 | (low adj3 middle adj3 countr*).ab,ti. | 17082 |
| 17 | (low* adj (gdp or gnp or (gross adj domestic) or (gross adj national))).ab,ti. | 248 |
| 18 | ((developing or (less* adj developed) or (under adj developed) or underdeveloped or (middle adj income) or (low* adj income)) adj (economy or economies)).ab,ti. | 569 |
| 19 | ((developing or (less* adj developed) or (under adj developed) or underdeveloped or (middle adj income) or (low* adj income) or underserved or (under adj served) or deprived or poor*) adj (countr* or nation or nations or population* or world or area or areas)).ab,ti. | 107787 |
| 20 | exp Asia/ | 837813 |
| 21 | exp West Indies/ | 26454 |
| 22 | exp South America/ | 162036 |
| 23 | exp Latin America/ | 11147 |
| 24 | exp Africa/ | 266871 |
| 25 | exp Developing Countries/ | 74898 |
| 26 | 9 or 10 or 11 or 12 or 13 or 14 or 15 or 16 or 17 or 18 or 19 or 20 or 21 or 22 or 23 or 24 or 25 | 1958344 |
| 27 | 8 and 26 | 1843 |
