# Supplementary material for: Strategies to adapt and implement health system guidelines and recommendations: a scoping review
Source: Health Res Policy Syst. 2022 Jun 15;20:64. doi: 10.1186/s12961-022-00865-8 (PMC9202131; doi:10.1186/s12961-022-00865-8)
Supplement: Supplementary file 2 — Additional file 2. Data extraction form. [file 12961_2022_865_MOESM2_ESM.docx]

Additional File 2: Data Extraction Form

| **Data Extraction Form** | |
| --- | --- |
| Year of Publication |  |
| Author (s) |  |
| Country (Context) |  |
| Type of Country Income |  |
| Study Aim(s) |  |
| Study Population |  |
| Setting |  |
| Funding |  |
| Intervention Name (Health System Guideline/Recommendation) |  |
| Guideline/Recommendation Description |  |
| Health System Building Blocks |  |
| Implementation/Adaptation Duration and Dose |  |
| Implementation Strategies (As per Proctor et al.) |  |
| Adaptation Strategies (As per FRAME) |  |
| Justification for implementation/ adaptation strategy |  |
| Stakeholder involvement |  |
| Outcomes of Interest |  |
| Study Methods |  |
| Barriers to Implementation/ Adaptation |  |
| Enablers to Implementation/ Adaptation |  |
| Key Results |  |
| Author Conclusions |  |
| Extractor Notes |  |
