# Supplementary material for: Strategies to adapt and implement health system guidelines and recommendations: a scoping review
Source: Health Res Policy Syst. 2022 Jun 15;20:64. doi: 10.1186/s12961-022-00865-8 (PMC9202131; doi:10.1186/s12961-022-00865-8)
Supplement: Supplementary file 3 — Additional file 3. Acronyms. [file 12961_2022_865_MOESM3_ESM.docx]

**Additional file 3: Acronyms**

| **Abbreviation** | **Definition** |
| --- | --- |
| ACCM | Attention to chronic conditions model |
| ANC | Atenatal care |
| ART | Antiretroviral therapy |
| BFCI | Baby friendly community initiative |
| BHU | Basic health units |
| CBM | Christian Blind Mission |
| CDC | Centres for Disease Control and Prevention |
| CFIR | Consolidated framework for implementation research |
| CHA | Community health assistant |
| CHMT | Council health management teams |
| CHW | Community health worker |
| CHX | Chlorhexidine |
| CRESIPT | Community randomized evaluation of a socioeconomic intervention to prevent TB |
| CUP | Contracting unit for primary care |
| DMO | District medical officer |
| DOTS | Directly observed treatment Short-course |
| DT | Dispersible tablets |
| EBF | Exclusive breastfeeding |
| EmOC | Emergency Obstetric Care |
| FHS | Family health strategy |
| FMS | Free maternity services |
| HCP | Healthcare provider |
| Hep B | Hepatitis B |
| HIV/AIDS | Human immunodeficiency virus/acquired immunodeficiency syndrome |
| HSS | Health Systems Strengthening Program |
| ICRM | Ideal clinic realisation and maintenance |
| IDSR | Integrated disease surveillance and response |
| IMCI | Integrated Management of Childhood Illnesses |
| IPTp | Intermittent preventive treatment of pregnant women |
| LBW | Low birth weight |
| LMICs | Low- and middle-income countries |
| MCE-IMCI | Multi-country evaluation of IMCI effectiveness, cost and impact |
| MD | Medical doctor |
| MHPs | Mental Health Practitioners |
| MIP | Malaria in pregnancy programs |
| MoH | Ministry of Health |
| MOHFW | Ministry of Health and Family Welfare |
| MPBP | More physicians for brazil program |
| MPDR | Maternal and perinatal death reviews |
| NCS | National quality core standards |
| NGO | Non-governmental organization |
| NHI | National health insurance |
| NHIS | National health insurance scheme |
| NTWC | National Technical Working Committee for Newborn Health |
| PHC | Primary health care |
| PHI | Popular health insurance |
| PHO | Provincial health office |
| PHSHT | Prenatal HIV, syphilis and hepatitis B testing |
| PMAQ | Program for Improving access and quality to primary care |
| PMS | Patient monitoring system |
| PMTCT | Prevention of mother to child HIV transmission |
| PPP | Public-private partnership |
| PSBI | Pneumonia and possible serious bacterial infection |
| PWP | Patients with psychosis |
| SMS | Municipal health secretary |
| TB | Tuberculosis |
| UHE-Ps | Urban health extension professionals |
| UK | United Kingdom |
| UNICEF | United nations children’s emergency fund |
| USAID | United States agency for international development |
| WHO | World Health Organization |
