# Supplementary material for: Strategies to adapt and implement health system guidelines and recommendations: a scoping review
Source: Health Res Policy Syst. 2022 Jun 15;20:64. doi: 10.1186/s12961-022-00865-8 (PMC9202131; doi:10.1186/s12961-022-00865-8)
Supplement: Supplementary file 4 — Additional file 4. Barriers to and enablers of implementation categorized by the COM-B framework (opportunities category). [file 12961_2022_865_MOESM4_ESM.docx]

| *Additional Tables: Barrier and Enablers to Implementation Categorized by the COM-B Framework (Opportunities Category)* | | |
| --- | --- | --- |
|  | COM-B: Opportunities | |
| Author (Year) | Physical | Social |
| Amaral et al. (2008) | - Smaller and more distant municipalities were more likely not to have the appropriate resources (Barrier) - Smaller population & greater distance from capital city (Barrier) - Introduced in municipalities with higher piped water coverage (Enabler) | - None reported |
| Andrade et al. (2017) | - Reorganization of diagnostic test supply (Barrier) - Dual private and public sector (bypassing the procedures and prevented adequate management) (Barrier) - Communication challenges/lack of thereof via MOH and CHA's supervisors (Barrier) | - None reported |
| Bergerot et al. (2017) | - None Reported | - Changes incorporated were well accepted by the health care team and by patients (Enabler) - Reorganized the service, facilitated interprofessional communication (Enabler) |
| Blanco-Mancilla (2011) | - As in the case of federal health care institutions, local authorities need to sign an agreement to contract services out to private provider (Barrier) - Refusal of the local government to sign up to it. In decentralized service delivery systems, local governments have full responsibility for implementing health policies (Barrier) - Some healthcare providers refused to offer voluntary abortion in Mexico (Barrier) - No performance monitoring or incentive programs in place - difficult to encourage the desired behavior for policy implementation (Barrier) - Low percentage of target population covered (Barrier) - Policy required certification to perform - The number of certified health professionals were unproportionate to the amount of service need (Barrier) - Lack of participation by medical associations in the policy process - disconnection between medical education and health policy. Medical schools are not addressing the needs nor providing human resources that are in tune with the current design of health policies (Barrier) - Increase access of services when engagement with private and third-party sectors (Enabler) | - Lack of support from implementers and beneficiaries (Barrier) - Decentralized health care system - responsibility lies with the state health departments and agreements are between both federal and local authorities. The burden on local authorities caused disagreements and conflict (Barrier) - Conflictual relationship between federal and local governments because of opposing ideas, values, and beliefs (Barrier) - Private clinic providers were more concerned about making a profit and their reputation and less concerned about the issues and implementing new policy (Barrier) - Doctors performing abortions were subjected to aggression such as disparaging comments and insults and created adverse working environments (Barrier) - Strong collaboration between the Ministry of Health and state health departments including the international academic community assisted reformation of a new Public Health coalition (Enabler) - Participation of international NGO's providing financial resources, generate and disseminate information, provide training, and engage in service provision (Enabler) - Engagement with Human Rights Commissions at both local and national levels was advantageous for accountability purposes (Enabler) - Individuals in charge of day to day activities dictated the behavior towards the policy due to their position at the front line of service (Enabler/Barrier) - Members of formal decision-making positions acted promptly when needed (Enabler) |
| Bryce at al. (2005) | - Staff turnover was a serious impediment to sustained implementation (Barrier) - Lack of stability in the coverage of health care workers (Barrier) - Lack of coordination with facility-based case management training (Barrier) - Basic Management Tools (Enabler) - Working group to plan for implementation (Enabler) | - None reported |
| Callaghan-Koru et al. (2020) | - No Pre-existing local source of resource (Barrier) - Unregulated private sector which is difficult to implement/ influence (Barrier) - Time consuming process (Barrier) - Health system structure as a bifurcated system - two directorates which meant duplication and separate plans, budgets, and logistics for information system (Barrier) - Barriers to accessing health facilities such as inadequate staff, high turnover rates and lack of supervision for quality assurance measures (Barrier) - Supply chain barrier (Barrier) - Strong engagement with partnerships and working group (Enabler) - Identifications of BFCI champions in community units (Enabler) - Engagement and use of a community person as a resource (Enabler) - Rapid national training coverage (Enabler) | - Cultural practices supported application of other substances, influencing the uptake of this policy (Barrier/Enabler) - Engagement of adolescent mothers (Enabler) |
| Carneiro et al. (2018) | - Physical location acted as a barrier to access physicians (Barrier) | - None reported |
| Costa et al. (2014) | - Weak relation between referrals and consultations (Barrier) - Higher financial costs to the health system (Barrier) - Excessive demand in the laboratories (Barrier) - Lack of requests can hinder diagnostic investigations, as well as point out inefficiencies in the system pertaining to its integrality and resolvability (Barrier) - Financial participation (Enabler) | - None reported |
| Ditlopo et al. (2011) | - None Reported | - None reported |
| Doherty et al. (2017) | - Shortages of supplies (Barrier) - Lack of computer equipment for data-entry within facilities (Barrier) - Major challenge regarding service delivery was decentralization of Option B+ services to lower level facilities (Barrier) - Shortages of human resources and lack of physical infrastructure (Barrier) - Dependent on external donor funding (Barrier/Enabler) - Discrepancies between planning and practices, and uneven regional implementation compromised implementation (Barrier) | - At community level, option B+ was implemented in a high-stigma environment, mentor mothers, local mothers living with HIV trained to counsel and support women newly enrolled had to disguise reason for home visits to avoid disclosure of patients HIV status (Enabler) |
| Ejeta et al. (2020) | - Lack of tracking series (Barrier) - More well thought out plan for resources (Barrier) - More people to complete visits were necessary (Barrier) - Creating more awareness of Information System (Barrier) - System that provided data to be updated more frequently (Barrier) | - None reported |
| Febir eta al. (2015) | - High workload resulted in health care workers not able to complete examinations in IMCI intervention (Barrier) - Ghana moved to a reimbursement of treatment cost via NHIS and they will not reimburse a facility if lab test doesn't show positive diagnosis (Barrier) - Supply chain was inadequate and experience of stock out (Barrier) - Lack of laboratory facilities (Barrier) - Lack of communication for experienced problems (Barrier) | - None reported |
| Gueye et al. (2016) | - Integration, decentralization and accountability (Enabler) - There were several countries with inadequate training program (Barrier) - Staff motivation is an important aspect of human resource capacity, and depends on a number of factors: working conditions, financial incentives, correct and prompt compensation, management of staff and possibilities for professional advancement (Enabler) - Funding and Human Resource availability (Barrier/Enabler) - Multisectoral collaboration and partnership (Enabler) - Funding and Human Resource Availability | - Political commitment (Enabler) - Multisectoral collaboration and partnership (Enabler) - Lack of accountability (Barrier) |
| Halpern et al. (2010) | - Need for extensive human resources (Barrier) - Having at least one focal person at each site to oversee the system (Enabler) - Monthly follow- up visits and ongoing support from stakeholders (Enabler) - On-site training must be provided on a continual basis, as staff turnover is common and is a threat to the sustainability (Enabler) | - None reported |
| Investigators of WHO LBW Feeding Study Group (2016) | - Need adequate human resource (Barrier) | - None reported |
| Kavle et al. (2018) | - Insufficient links between community and hospital level resulting in inadequate implementation, knowledge gaps and follow up (Barrier) - Insufficient number of BFCI master trainers (Barrier) - Inadequate coverage for entire country (Barrier) - Lack of community units for training (Barrier) - Lack of allocation of funds (Barrier) - Insufficient number of counseling cards (Barrier) - Inadequate physical space to plant gardens to promote nutrition (Barrier) - Difficulty following up of mothers due to migration of mothers residing in informal settlements (Barrier) - Health Worker strike (Barrier) - The transfer of trained health workers to other health facilities leads to a gap in provider capacity to implement in new facilities (Barrier) - Poor attendance in the support group sessions (Barrier) - Large number of partnerships (Enabler) - Building of new community centers (Enabler) - Development of national guidance and materials to support implementation roll out and training (Enabler) - Integration with other sectors including Agriculture linkages, Linkage with the Ministry of Education, linkage with support groups to provide income generation (Enabler) - New system for real – time documentation (Enabler) - Monitoring of 5 key BFCI indicators (Enabler) - Support of BFCI by political administering and politicians (Enabler) - Community ownership to ensure that the community took lead in the process to ensure stability of implementation (Enabler) - Engagement and use of community’s own resource persons (Enabler) - Engagement and early recruitment of participants (Enabler) | - Political unrest and instability, affecting attendance of community support groups (Barrier) - Mothers who joined the support group were supported from the start and had improved attendance and showed stability (Enabler) |
| Kihembo et al. (2018) | - Lessons from Ebola response allowed buy in and acceptance of ISDR strategy implementation (Enabler) - Funding was included in budgets (Enabler) - Implementation was poorly conducted which led to weak integration of some activities into existing maternal health services (Barrier) | - Training brought together health workers of multiple disciplines and they appreciated knowledge of other (Enabler) |
| Lavar et al. (2016) | - Technical, political, and financial autonomy (Barrier) - Availability of laboratory resources (Enabler) | - None reported |
| Leethongdee (2007) | - A bill was introduced that the MoPH retain MoPH retained responsibility for budget allocation for three years, this led to the PHOs were buying services from hospitals which they also directly managed, and continued to be a source of tension as implementation proceeded (Barrier) - Health system reform required additional building capacity and a new financial system at the provincial level (Barrier) - Allocation of financial resources to hospitals were unevenly dispersed at the macro and micro level:   a) Income inequality and income distribution issues in the local, micro level b) Failure of the government to pay the full amounts (especially to smaller hospitals) at the national, macro level (Barrier)   - Lack of physicians per capita - difficult to recruit. Unfavorable ratio of health care facilities and professional staff to population (Barrier) - Planning, coordination functions on the purchaser side was underdeveloped (Barrier) - Burden of work on the health care staff and lack of reward (Barrier) - Developing a system for evaluating health service bodies against key performance indicators (Enabler) - Good channels of communication and strong relationship between key departments of government agencies (Enabler) | - Imbalance of power - Community providers in rural areas were under control of the doctor directors of community hospitals and used their power to allocate financial resources according to their own priorities (Barrier) - Weak relationship between District Health Office and hospitals (Barrier) - Need for greater public participation and engagements (Barrier) - Organizational reform created tension between the old and new administrative structure (Barrier) - Imitation of an ad hoc working group to monitor and troubleshoot emergent problems associated with the reform of the health system (Enabler) |
| Li et al. (2015) | - Ability to distribute drugs (Barrier) - Lack of appropriate laws and regulations to regulate essential drug manufacturing (Barrier) | - None reported |
| Lovero et al. (2019) | - Absence of coordination across health programs in district level administration (Barrier) - Lack of material and human resources (Barrier) | - None reported |
| Miguel-Esponda et al. (2020) | - Lack of mental health training for MDs (Barrier) - Limited time due to the numerous tasks that providers are responsible for (resource limiting) (Barrier) - Large patient loads at the clinics (Barrier) - Appointment of a programme coordinator and a team that promoted the delivery of mental health services and provided continuous support to do this (Enabler) - Funds for the purchase of medications (Enabler) - Support structure provided by CES makes the delivery of services possible (Enabler) | - Programme was also largely acceptable to providers (Enabler) |
| Mkoka et al. (2014) | - A shortage of skilled health workers (Barrier) - Financial constraints and delayed disbursement of funds (Barrier) - Lack of incentives to motivate overburdened staffs (Barrier) - Lack of guidelines for partnership development (Barrier) - Governance issues at different levels - health facility governing committee were reported to be weak (Barrier) - Unclear mechanisms for accountability (Barrier) - NGOs and development partners supplemented resource gaps by supplying facilities with equipment needed for emergency obstetric care (Enabler) | - CHMT worked closely, not only with the central government, but also with other partners found in the district, including local government officials, development partners and NGOs (Enabler) |
| Moshiri et al. (2016) | - Incomplete network coverage in urban areas (Barrier) - Lack of a functional referral system (Barrier) - The limited nature of collaborations between the Deputy for Health and Deputy for Treatment Affairs (Barrier) - The role of some international organizations in support of the Iranian PHC in the international meetings (Enabler) - Shortages of doctors in rural areas (Barrier) - Government provided financial support for the establishment of PHC network with the condition that there is continuation of the program (Enabler) | - Political atmosphere in the country sometimes made restrictions on the activities (Barrier) - Prior engagement of policy designers with location actors shaped a friendly and collaborative change environment (Enabler) - Pressure on the government to provide social services for all citizens improved access to health care services/programs (Enabler) |
| Mutabazi et al. (2020) | - Bureaucratic slowness (Barrier) - Unsupportive Department of Health, difficulty obtaining approvals at local clinics (Barrier) - Small working spaces and other infrastructure challenges (Barrier) - Staff shortages and work overload (Barrier) - Lack of national health information systems and issues relating to electronic records (Barrier) - Staff shortages and work overload (Barrier) - High staff turnovers (Barrier) - Changes in guidelines (Barrier) - Too much paperwork for managers (Barrier) - Integration of PMTCT services in local health facilities benefited other services (Enabler) - NGO's helped from the beginning though providing support and follow up with women in community (Enabler) | - Discrimination/stigma (Barrier) |
| Muthathi et al. (2020) | - Insufficient clarity on the roles and responsibilities - weak intergovernmental relationships (Barrier) - Unfunded mandates (Barrier) - Lack of accountability to communities (Barrier) - Disjuncture between two national quality of care initiatives and resource constraints influenced policy actors’ experiences and perceptions (Barrier) - Local leadership enabled implementation - experienced and knowledgeable managers were able to motivate or negotiate for additional resources to accelerate implementation (Enabler) | - Frustrations about the lack of or diffuse accountability and stakeholders lack of involvement in decision making (Barrier) |
| Pyone et al. (2017) | - Compromised by operational challenges, including delay in receiving reimbursements at health facilities, shortages of health workers, drugs and supplies (Barrier) - Gaps in policy dissemination and information asymmetry (Barrier) - Lack of clarity about the new policy (Barrier) - Weak enforcement mechanisms (e.g., delayed reimbursement to health facilities, which led to continued levying of service charges) (Barrier) - Misaligned incentives (Barrier) - Reluctant to pay for maternity care using their insurance as they assumed the services were free for all (Barrier) - Ensuring the basic needs of health workers (receiving sufficient salary on time) was crucial for motivating them to carry out their responsibilities (Enabler) - Successful institutional coherence (Enabler) - Reform incentives were consistent with clear mandates from all line ministries and intense political pressure to comply with the reform (Enabler) | - None reported |
| Rahman et al. (2020) | - Insufficient coordination among government sectors (Barrier) - Lack of central procurement of amoxicillin DT (Barrier) - Some HSP didn't have respiratory counter (Barrier) - Some HSP didn't have authority to treat (Barrier) - Some caregivers had hard time administrating and measuring medication (Barrier) - Lack of access to the pneumonia guidelines (Barrier) | - None reported |
| Roman et al. (2014) | - Commodities - lack of resources and stock which dampened uptake of MIP interventions (Barrier) - Ineffective distribution system (Barrier) - Lack of funding (Barrier) - Lack of competing responsibilities MOH (Barrier) - Lack of human resources, and staff caused inconsistent patient visits also treatments were inconsistent (Barrier) - Lack of funding overall, health workers not being paid etc. (Barrier) | - Community involvement (Enabler) |
| Ryan et al. (2020) | - None Reported | - NGOs play a particular important role in channeling resources to mental health in LMICs |
| Saddi et al. (2018) | - Resistance of professionals (Barrier) - Organizational context of the primary health care (with a lacking/fragile culture of evaluation and excessive workloads) affects the health worker involvement and their perceptions (Barrier) - Influence of financial incentives is partial or requires adaptations/changes to take place (Barrier) - How the program is developed in complex scenarios, in which health workers must cope with several demands (investments in the organizational capacity of the unit would foster and facilitate workers’ participation in the PMAQ) (Enabler) - Lack of guidelines for partnership development (Barrier) | - Lack of support or guidance (Barrier) |
| Sami et al. (2018) | - Lack of policy support inhibited ability to expand implementation to other sites or plan for activities (Barrier) - Shortage of workers and many of them were new grads which required additional training on the job (Barrier) - Lack of documentation and too time consuming to complete (Barrier) - Lack of transportation (Barrier) - Availability of essential medical commodities for newborn care (Enabler) | - Positive interaction between caregivers and health worker (Enabler) |
| Schneider & Nxumalo (2017) | - Fragmented policy - different interpretations (Barrier) - Ambiguity in the national WBOT strategy (Barrier) - Not backed up by proper funding (restructuring requires significant investments) (Barrier) - Distributed nature of the leadership and governance functions (Barrier) - Development of policies required flexibility as it’s a dynamic, negotiate, iterative process (Enabler) | - Building a political commitment and achieving consensus on a different orientation (Enabler) |
| Sheikh et al. (2010) | - Lack of support from superior authorities in combination with the lack of authority over the behavior of changing medical providers clinical judgement (Barrier) - Physical task of regulating providers was beyond the resources and means of the program (Barrier) | - Lack of dialogue between the right actors - imbalance of power (Barrier) - Divergences in ethical orientation between culturally different doctors (Barrier) |
| Shelley et al. (2016) | - Community Acceptance- Too many patients, not enough CHA led to difficulties with time management and patients left without being seen  Supervision Support System (Barrier) - Lack of transport, work overload, and travel distance (Barrier) - Health System Challenges: Delay in CHA's receiving monthly salaries (Barrier) - Lack of drug resources via national supply chain (Barrier) | - None reported |
| Stein et al. (2008) | - The nurses at the clinic were also still over-stretched and many PHC clinics are chronically under-staffed | - The ongoing support and engagement of nurses and trainers was a huge advantage - they provided the support that clinic-level managers didn't supply (Enabler) |
| Wingfield et al. (2015) | - Account maintenance charges were introduced during implementation and impact trust (Barrier) - Access to the bank provider (Barrier) | - High risk patients in more urban communities were difficult to engage with (Barrier) |
| Xia et al. (2015) | - Human Resources - Not enough staff trained (Barrier) - Lack of uniform test counselling technology guidance and training for providing these services (Barrier) - Funds were not adequate, and the lab infrastructure didn't receive any additional funding so lack of motivation to complete test (Barrier) - Lack of communication of test results through the integration of Information system led to overlap of work (Barrier) - Lack of mechanism for coordinated management (Barrier) - Lack of the referral and communication networks with agreed guidelines (Barrier) - Roles and responsibilities of different health agencies were unclear (Barrier) - Outcome evaluation systems were not integrated among the different health agencies (Barrier) - Referrals and results took too long to process (Barrier) - Lack of communication between the 4 health agencies (Barrier) | - None reported |
| Zhou et al. (2019) | - Insufficient and poorly trained human resources (Barrier) - Funding without guarantee (Barrier) - Policy content had operational defects of lacking necessary details on evaluation, funding and activity (Barrier) - Discrepancies between planning and practices, and uneven regional implementation (Barrier) | - Low acceptability of service recipients (Barrier) |

| *Additional Tables: Barrier and Enablers to Implementation Categorized by the COM-B Framework (Capabilities Category)* | | |
| --- | --- | --- |
|  | Capability | |
| Author (Year) | Psychological | Physical |
| Amaral et al. (2008) | - None reported | - None reported |
| Andrade et al. (2017) | - None reported | - None reported |
| Bergerot et al. (2017) | - Considering the available evidence and the standards proposed in consensus by different experts (Enabler) | - On-site training must be provided on a continual basis, as staff turnover is common and has proven to be one of the main threats to the sustainability (Barrier/Enabler) |
| Blanco-Mancilla (2011) | - Poor understanding of policies (Barrier) | - None reported |
| Bryce at al. (2005) | - None reported | - Inability to sustain supervision systems to overcome distance and transportation barriers - Increased documentation and supervision (Enabler) |
| Callaghan-Koru et al. (2020) | - None reported | - Simple intervention that was easily taught and accepted by providers and families (Enabler) |
| Carneiro et al. (2018) | - None reported | - Educational actions (Enabler) |
| Costa et al. (2014) | - None reported | - None reported |
| Ditlopo et al. (2011) | - None reported | - None reported |
| Doherty et al. (2017) | - None reported | - None reported |
| Ejeta et al. (2020) | - Some households were unwilling to provide information (Barrier) | - Handling of too many cards was challenging (Barrier) |
| Febir eta al. (2015) | - None reported | - Few health care workers have been trained and untraining people weren’t able to perform assessments (Barrier) |
| Gueye et al. (2016) | - None reported | - Strong supervision |
| Halpern et al. (2010) | - None reported | - On-site training must be provided on a continual basis, as staff turnover is common and has proven to be one of the main threats to the sustainability (Barrier/Enabler) |
| Investigators of WHO LBW Feeding Study Group (2016) | - Mothers require some time and persistent motivation before they can translate their improved knowledge into practice (Enabler) - The health providers had a fair degree of knowledge and skills pertaining to care of LBW babies at baseline (Enabler) | - None reported |
| Kavle et al. (2018) | - Lack of knowledge causing insufficient links within the community (Barrier) | - Lack of follow up support causing insufficient links within the community (Barrier) - No national training curriculum tailored for community-based providers (Barrier) - Difficulty follow up on participants due to migration of mothers physically relocating, and arid settlements (Barrier) - Identification of champions in community units (Enabler) - Continued implementation through training mentorship, supportive supervision and follow up with documentation (Enabler) |
| Kihembo et al. (2018) | - Training brought together health workers of multiple disciplines and they appreciated knowledge of other departments contribution (Enabler) | - Training program equipped all health workers trained with SOP's guidelines, case definitions, manuals, reporting forms guidelines (Enabler) - Training laboratory workers health supplement the efforts to strengthen its capacity (Enabler) - DRRT training of multi-disciplinary team promoted readily mobilized and deployment for rapid outbreak (Enabler) - Training approach of a combination of cascade effect and only building on their specific skill set didn't burden their time away from their daily job and strain the system (Enabler) |
| Lavar et al. (2016) | - None reported | - None reported |
| Leethongdee (2007) | - None reported | - Lack of confidence in the capabilities of health center staff (Barrier) |
| Li et al. (2015) | - None reported | - Township hospitals have organized the trainings of basic drugs use for doctors (Enabler) |
| Lovero et al. (2019) | - Low mental health awareness in both the district administration and general population (Barrier) | - None reported |
| Miguel-Esponda et al. (2020) | - None reported | - MDs reported that training sessions were helpful but insufficient to develop the skills that mental health consultations require (Barrier) |
| Mkoka et al. (2014) | - None reported | - None reported |
| Moshiri et al. (2016) | - Lack of knowledge of how to store vaccines resulted in vaccines being expired and ineffective (Barrier) | - National planners made unexpected visits to health centers in a serious stringent manner to ensure program is being administered (Enabler) |
| Mutabazi et al. (2020) | - Resistance to change; NGO's helped from the beginning though awareness campaigns (Barrier) | - Poor training: NGO's helped from the beginning though, assisting in the training (Enabler/Barrier) |
| Muthathi et al. (2020) | - None reported | - None reported |
| Pyone et al. (2017) | - None reported | - None reported |
| Rahman et al. (2020) | - None reported | - None reported |
| Roman et al. (2014) | - None reported | - None reported |
| Ryan et al. (2020) | - None reported | - None reported |
| Saddi et al. (2018) | - Font-liner workers do not hold a high level of knowledge (Barrier) | - None reported |
| Sami et al. (2018) | - Failure to prioritize newborn activities among program supervisors (Barrier) | - Protocols were challenging and required a higher level of health worker to attend. Simpler protocols would have allowed nurses to take on additional responsibilities (Barrier) - Lack of technical skills resulted in failing to apply for funding properly (Barrier) |
| Schneider & Nxumalo (2017) | - None reported | - None reported |
| Sheikh et al. (2010) | - Disparities of meanings in the implementation actors (Barrier) - Hospital administrators didn't perform their roles in regulatory functions to ensure the implementation (Barrier) - Lack of engagement to create meaningful dialogue between government departments and providers (Barrier) | - None reported |
| Shelley et al. (2016) | - None reported | - Some expressed more training on health topics would be beneficial (Enabler) - Clinical supervision (Enabler) |
| Stein et al. (2008) | - Increase Workload of positive HIV patients (very ill patients) had a high emotional toll on nurses (Barrier) - The onsite training created a disadvantage by nurses experiencing the pressure of their patients waiting for their attention (Barrier) | - None reported |
| Wingfield et al. (2015) | - None reported | - Provided community meetings consisting of educational workshops (Enabler) |
| Xia et al. (2015) | - Lack of knowledge of services (Barrier) | - Very few health care workers have been trained in IMCI and untrained people weren't able to carry out assessments (Barrier) |
| Zhou et al. (2019) | - Insufficient and poorly trained human resources (Barrier) | - None reported |

| *Additional Tables: Barrier and Enablers to Implementation Categorized by the COM-B Framework (Motivation Category)* | | |
| --- | --- | --- |
|  | Motivation | |
| Author (Year) | Automatic | Reflective |
| Andrade et al. (2017) | - None reported | - Roles of Community Health Authorities and the Health managers are fundamental in the PHC network (Enabler) |
| Blanco-Mancilla (2011) | - None reported | - Given the sensi1tivity of policy (voluntary abortion), the negative idea and beliefs that implementers had about the policy hindered implementation (Barrier) - In general, the negative perceptions about the PHI policy made it difficult to promote (Barrier) |
| Callaghan-Koru et al. (2020) | - None reported | - Did not infringe on clinical scope of practice and little controversy (Enabler) |
| Costa et al. (2014) | - Patient anxiety (Barrier) |  |
| Ejeta et al. (2020) | - None reported | - More clear definition of roles and responsibilities of stakeholders (Barrier) |
| Febir eta al. (2015) | - None reported | - Lack of trust in the accuracy of RDT testing measures (Barrier) |
| Gueye et al. (2016) | - None reported | - Staff had too many roles and responsibilities that were not clearly defined (Barrier) |
| Kavle et al. (2018) | - Lack of motivation (unpaid workers) (Barrier) | - Champions in the community (Enabler) |
| Kavle et al. (2018) | - Lack of motivation/engagement due to lack of reasoning to enforce the guidelines amongst their staff (Barrier) | - None reported |
| Leethongdee (2007) | - Community stakeholders were less supportive in promotion projects and more into projects that would develop local infrastructure (Barrier) | - Confusion in the line of command at the district level (Barrier) |
| Rahman et al. (2020) | - Perceptions of the efficacy of antibiotics and formulations at the national and district levels (Barrier/Enabler) | - None reported |
| Sami et al. (2018) | - The motivation package for malaria workers, structure of incentives, training programmes, and supervisory structure offered by malaria control programmes (Enabler) - Maintaining a high level of motivation in implementers is important to sustain consistency and quality of interventions (Enabler) | - None reported |
| Sheikh et al. (2010) | - None reported | - Different ethical orientation in terms of informed consent (Barrier) - Practitioners don't agree with the change (Barrier) - Tasks in the guideline were seen as impediments and secondary in nature (Barrier) - Medical professionals resist change and authority in all forms along with no reason for them to enforce the guidelines and administrative appeared to express no authority over practitioners (Barrier) |
| Stein et al. (2008) | - Enthusiasm for newly commenced roll outs (Enabler) | - None reported |
| Wingfield et al. (2015) | - Delays in cash transfers eroded participants’ trust in the project and stigma (Barrier) | - None reported |

| *Additional Tables: Capabilities Opportunities Motivation Summary Table (Barriers and Enablers to Implementation)* | | | | | | | |
| --- | --- | --- | --- | --- | --- | --- | --- |
|  | Opportunity | | Capabilities | | Motivation | |  |
| Author (Year) | Physical | Social | Psychological | Physical | Automatic | Reflective | Total |
| Amaral et al. (2008) | X |  |  |  |  |  | 1 |
| Andrade et al. (2017) | X |  |  |  |  | X | 2 |
| Bergerot et al. (2017) |  | X | X | X |  |  | 3 |
| Blanco-Mancilla (2011) | X | X | X |  |  |  | 3 |
| Bryce at al. (2005) | X |  |  | X |  |  | 2 |
| Callaghan-Koru et al. (2020) | X | X |  | X |  | X | 4 |
| Carneiro et al. (2018) | X |  |  | X |  |  | 2 |
| Costa et al. (2014) | X |  |  |  | X |  | 2 |
| Ditlopo et al. (2011) |  |  |  |  |  |  | 0 |
| Doherty et al. (2017) | X | X |  |  |  |  | 2 |
| Ejeta et al. (2020) | X |  | X | X |  | X | 4 |
| Febir eta al. (2015) | X |  |  | X |  | X | 3 |
| Gueye et al. (2016) | X | X |  | X | X |  | 4 |
| Halpern et al. (2010) | X |  |  | X |  |  | 2 |
| Investigators of WHO LBW Feeding Study Group (2016) | X |  | X |  |  |  | 2 |
| Kavle et al. (2018) | X | X | X | X | X | X | 6 |
| Kihembo et al. (2018) | X | X | X | X |  |  | 4 |
| Lavar et al. (2016) | X |  |  |  |  |  | 1 |
| Leethongdee (2007) | X | X |  | X |  |  | 3 |
| Li et al. (2015) | X |  |  | X |  |  | 2 |
| Lovero et al. (2019) | X |  | X |  |  |  | 2 |
| Miguel-Esponda et al. (2020) | X | X |  | X |  |  | 3 |
| Mkoka et al. (2014) | X | X |  |  |  |  | 2 |
| Moshiri et al. (2016) | X | X | X | X |  |  | 4 |
| Mutabazi et al. (2020) | X | X | X | X |  |  | 4 |
| Muthathi et al. (2020) | X | X |  |  |  |  | 2 |
| Pyone et al. (2017) | X |  |  |  |  |  | 1 |
| Rahman et al. (2020) | X |  |  |  | X |  | 2 |
| Roman et al. (2014) | X | X |  |  |  |  | 2 |
| Ryan et al. (2020) |  | X |  |  |  |  | 1 |
| Saddi et al. (2018) | X | X | X |  |  |  | 3 |
| Sami et al. (2018) | X | X | X | X |  |  | 4 |
| Schneider & Nxumalo (2017) | X | X |  |  |  |  | 2 |
| Sheikh et al. (2010) | X | X | X |  |  | X | 4 |
| Shelley et al. (2016) | X |  |  | X |  |  | 2 |
| Stein et al. (2008) | X | X | X |  |  | X | 4 |
| Wingfield et al. (2015) | X | X |  | X | X |  | 4 |
| Xia et al. (2015) | X |  | X | X |  |  | 3 |
| Zhou et al. (2019) | X | X | X |  |  |  | 3 |
| Total | 36 | 22 | 15 | 19 | 4 | 10 |  |

| *Additional File: Capabilities Opportunities Motivation Summary Table (Barriers and Enablers to Adaptation)* | | | | | | | |
| --- | --- | --- | --- | --- | --- | --- | --- |
|  | Opportunities | | Capabilities | | Motivation | |  |
| Author (Year) | Physical | Social | Psychological | Physical | Automatic | Reflective | Total |
| Andrade et al. (2017) | X |  |  |  |  |  | 1 |
| Bryce at al. (2005) | X |  |  |  |  |  | 1 |
| Gueye et al. (2016) | X |  |  |  |  |  | 1 |
| Halpern et al. (2010) | X |  |  |  |  |  | 1 |
| Leethongdee (2007) | X |  |  |  |  |  | 1 |
| Rahman et al. (2020) | X | X |  |  |  |  | 2 |
| Stein et al. (2008) |  | X |  |  |  | X | 2 |
| Wingfield et al. (2015) |  | X |  |  |  | X | 2 |
| Total | 6 | 3 | 0 | 1 | 0 | 2 |  |
